# Bioinspired synthesis of a lactam analogue of abyssomicin C^☆^

**DOI:** 10.1016/j.tetlet.2025.155778

**Published:** 2025-08-06

**Authors:** A. Cole Edwards, Joshua G. Pierce

**Affiliations:** Department of Chemistry, Comparative Medicine Institute, and Integrative Sciences Initiative, NC State University, Raleigh, NC 27607, USA

**Keywords:** Natural products, Diels alder, Biomimetic, Antibiotics

## Abstract

The bioinspired approach to abyssomicin C developed by Sorensen has been utilized to prepare a novel analog containing an α, β-unsaturated lactam. This synthesis was designed to utilize the intramolecular Diels-Alder reaction in late-stage synthesis to provide analogs that contain deep-seated modifications to explore their antimicrobial properties. Overall, installing this non-natural lactam moiety was successful and allowed for antimicrobial assessment of the analog, but the low yields highlight the frequent challenges of leveraging a bioinspired approach for a new-to-nature molecular target.

Abyssomicin C (**1,** Fig. 1), first isolated from the Japanese sea in 2004 by Süssmuth and co-workers, is a member of the larger class of spirotetronate marine natural products [1,2]. In addition to the complex spirotetronate core, the abyssomicins exist as interconverting mixtures of atropisomers under mildly acidic or physiological conditions. Initial investigations of the antibacterial properties of abyssomicin C revealed potent activity against Gram-positive bacteria, including multidrug-resistant strains of *S. aureus* (5–13 μg/mL). The antimicrobial activity of this compound originates from an inhibition of the chorismate pathway, which leads into the folate pathway [1]. This pathway is notably absent from human and other mammal metabolic processes, making targeting this pathway an attractive approach for drug design. Abyssomicin C affects bacterial folate biosynthesis through a covalent deactivation of a subunit of 4-amino-4-deoxychorismate (ADC) synthase, the enzyme responsible for the conversion of chorismate and glutamine into ADC and glutamate. Thia-Michael addition of a cysteine residue to the enone moiety present in abyssomicin C.

leads to the irreversible formation of a covalently bound enzyme adduct [3]. A covalent mechanism is supported by the lack of activity for abyssomicin H (**2**) wherein the enone moiety is reduced to the corresponding ketone (Fig. 1). While the natural product shows promise as an antimicrobial lead, there remains significant questions about selectivity, potency, and ultimate in-vivo efficacy. This is highlighted by studies from Sorensen and co-workers that revealed nanomolar potency of atrop-abyssomicin C (**1a**) against mammalian cell lines (Fig. 1) [4].

The novel and complex structure and promising antimicrobial activity drove interest from the synthetic community in the years following the isolation of **1**. To this end, several total syntheses of **1** and related derivatives were reported in the past several decades [5–11]. A tactic common to multiple syntheses of this natural product is the use of a biomimetic [4 + 2] reaction to form the central spirotetronate core, highlighted by Sorensen’s use of a late-stage, biomimetic transannular Diels-Alder reaction to prepare abyssomicin C from a linear precursor [5]. To date, there are no reports of naturally occurring or synthetic derivatives of **1** that have subtly modified electronics or sterics of the enone moiety. The only insight into the impact of enone electrophilicity is in the case of the atropisomer **1a**, wherein the geometry of the medium-sized ring is altered, allowing for greater overlap of the π-system of the enone [8]. This compound has demonstrated increased electrophilicity in chemical addition reactions, yet only shows subtle differences in biological activity [8]. Herein, we report our efforts to modify the structure of the medium-sized ring, replacing the α,β-unsaturated ketone with an α,β-unsaturated amide by taking advantage of the late-stage Diels-Alder reaction utilized first for the natural product by Sorensen and Snider [5,6]. This effort is part of our larger program targeting deep-seated structural modifications of **1** and developing next-generation antibiotic lead scaffolds [12,13].

While structurally distinct from **1**, the retrosynthetic analysis of lactam abyssomicin **3** (Fig. 2) was envisioned to closely mirror that reported by Sorensen [5]. Installation of the secondary alcohol in **3** would arise from an intramolecular epoxide ring opening using the appended tetronate as the intramolecular nucleophile. This nucleophile would be generated from a Krapcho-type demethylation of the corresponding vinylogous methyl carbonate. The epoxide would be generated from the epoxidation of a cyclohexene which would be furnished from an intramolecular Diels-Alder reaction of **4**. This system would be subtly different in electronics, as the electron density of the 2,4,6-octatrienamide would be greater than that of the ketone variant previously reported for abyssomicin C. The tethered diene and dienophile for this cycloaddition would be prepared from the lithiation of the known methyl tetronate derivative **6** and the corresponding aldehyde. This aldehyde would be broken down to 2,4,6-octatrienoic acid and β-alanine methyl ester, the former from hexadienal (**7**) and the latter from β-alanine (**5**). This amino acid was chosen to remove chirality in the starting material in hopes of simplifying the initial synthesis of the targeted analog.

In a forward sense, hexadienal (**7**) was converted to the known trienoic acid **8**^5^ using the Doebner modification of the Knoevenagel condensation (Scheme 1). This carboxylic acid was then used to acylate β-alanine methyl ester **9**, generated in one step from **5**, resulting in secondary amide **10** in good yield and on a gram-scale. Amide **10** was subsequently methylated using sodium hydride as a base to provide **11** in modest yield. This reduced yield appeared to be due to over-methylation and elimination byproducts. The ester in **11** was then reduced to the alcohol using lithium borohydride as the reducing agent. The resulting primary alcohol was oxidized to the aldehyde **13** under Swern conditions. We used Swern oxidation in this step due to more consistent conversion and cleaner reaction profiles compared to DMP, which led to decomposition in our hands. It is important to note that a mixture of alkene isomers is carried through this process, and that amide rotamers are prevalent in the spectra of these compounds.

With **13** in hand, it was now required to install the tetronate moiety through a lithiation-addition reaction (Scheme 2). This reaction has been used extensively throughout the literature [4–7,11], employing the well-known methyl tetronate derivative and lithium diisopropylamide to form the corresponding organolithium reagent. This reaction usually proceeds with poor to moderate yields in the literature and has been shown to require temperatures lower than − 78 °C to control reproducibility [4,11]. These challenges were also observed in the present case, with the reaction requiring −95 °C. The crude product **14** was found to be best handled by carrying it on after limited purification. The secondary alcohol product from this addition was then oxidized to the ketone using Dess-Martin periodinane to provide key intermediate **4** that was then used without purification in the intramolecular Diels-Alder to yield **15** in 13 % yield over 3 steps. This cycloaddition was diastereoselective like those achieved by Sorensen and Snider, but not enantiospecific as these intermediates lack the methyl groups present in **1**. We considered utilizing the alcohol directly in this reaction, but were not successful in early attempts. While the yields of this reaction are lower than that of the natural product, we attribute these challenges to the lack of preorganization in the present case, as well as the alteration in the conformation of the amide linkage in **4**.

With **15** in hand, it was time to execute the final epoxidation/epoxide opening sequence, which has become the most common approach to assembling the spirotetronate moiety. A roadblock that wasn’t expected at this junction was the selective epoxidation of the cyclohexene over the alkene of the α,β-unsaturated amide. Using DMDO and *m*CPBA, epoxidation of both alkenes occurred, generating three different products. Shi epoxidation conditions as well as VO(acac)_2_ resulted in the decomposition of the starting material. Under the optimal conditions (*m*CPBA, 0 °C) the target compound **16** was isolated as a mixture of two isomers (~1:1 regioisomers + other trace stereoisomers) by NMR, which was carried forward as a mixture. The vinylogous carbonic acid **17** was then revealed under Krapcho-type conditions and this product was subsequently subjected to (1*S*)-(+)-10-camphorsulfonic acid in the final step to form the targeted analog **3** through intramolecular ring-opening of the epoxide. Only the desired epoxide proceeds through this ring-opening step, thereby allowing for the isolation of this compound more readily. The structure of **3** was further confirmed through X-Ray analysis (see Supporting Information).

At this stage, we sought to evaluate the activity of **3** against methicillin-resistant *Staphylococcus aureus* (MRSA), serving as a probe as to the impact of modulated electrophilicity on the enone warhead of these natural products. Unfortunately, the minimum inhibitory concentration was collected against two strains of MRSA (ATCC 43300 and ATCC 33591), and both showed an MIC >128 μg/mL. Examination of the X-ray crystallographic data shows a bond angle deviation from atrop-abyssomicin C (Fig. 3). The α,β-unsaturated ketone in atrop-abyssomicin C (**1a**) rests in a cis-oid configuration instead of trans-oid like abyssomicin C [8]. This cis-oid configuration is also taken in the synthetic lactam-abyssomicin **3**, but with almost a 15° deviation further away from planarity of the electronic system responsible for microbial activity compared to **1a**.

In conclusion, a synthetic, deep-seated structural modification to the abyssomicin skeleton has been achieved, editing a functional group responsible for its known antimicrobial activity. A biomimetic intramolecular Diels-Alder reaction allowed diastereoselective access to the carbon skeleton as seen in the natural product, but late-stage epoxidations resulted in poor regioselectivity. While biomimetic synthetic strategies can be compelling for access to natural products themselves, targeting analogs with these approaches can lead to challenges in yield and selectivity. Efforts are currently underway to develop abiotic synthetic approaches to a library of derivatives to further explore the biological activity of the abyssomicin family of natural products and develop probes to explore their selectivity and activity. These studies will be reported in due course.

## Supplementary Material

1

## Figures and Tables

**Fig. 1.**
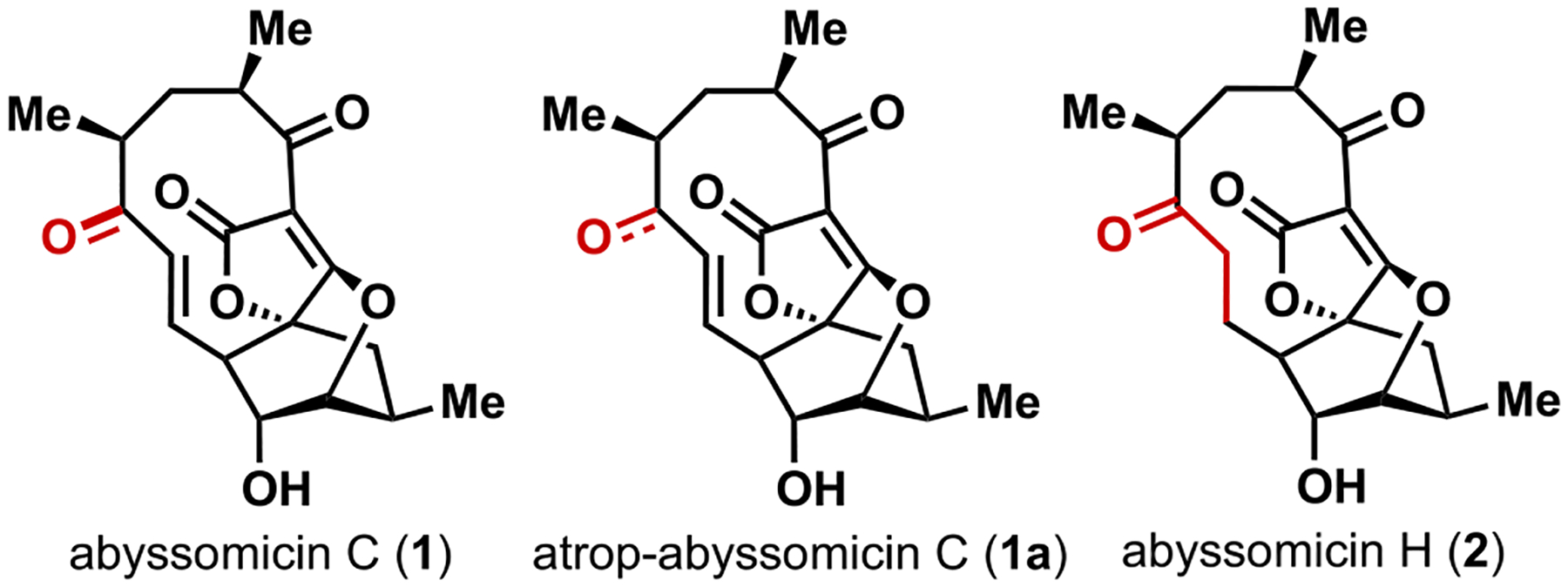
Abyssomicin natural products.

**Fig. 2.**
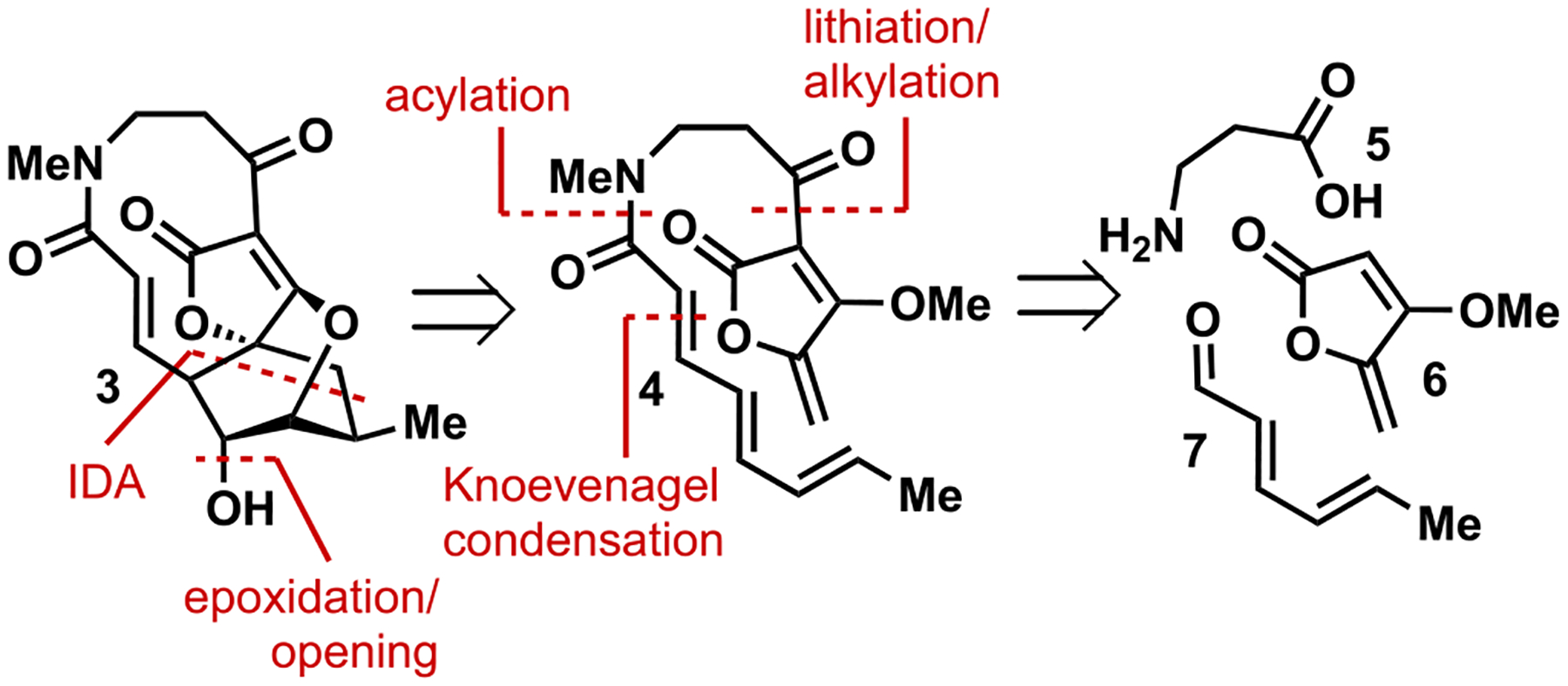
Retrosynthetic strategy toward lactam **3**.

**Fig. 3.**
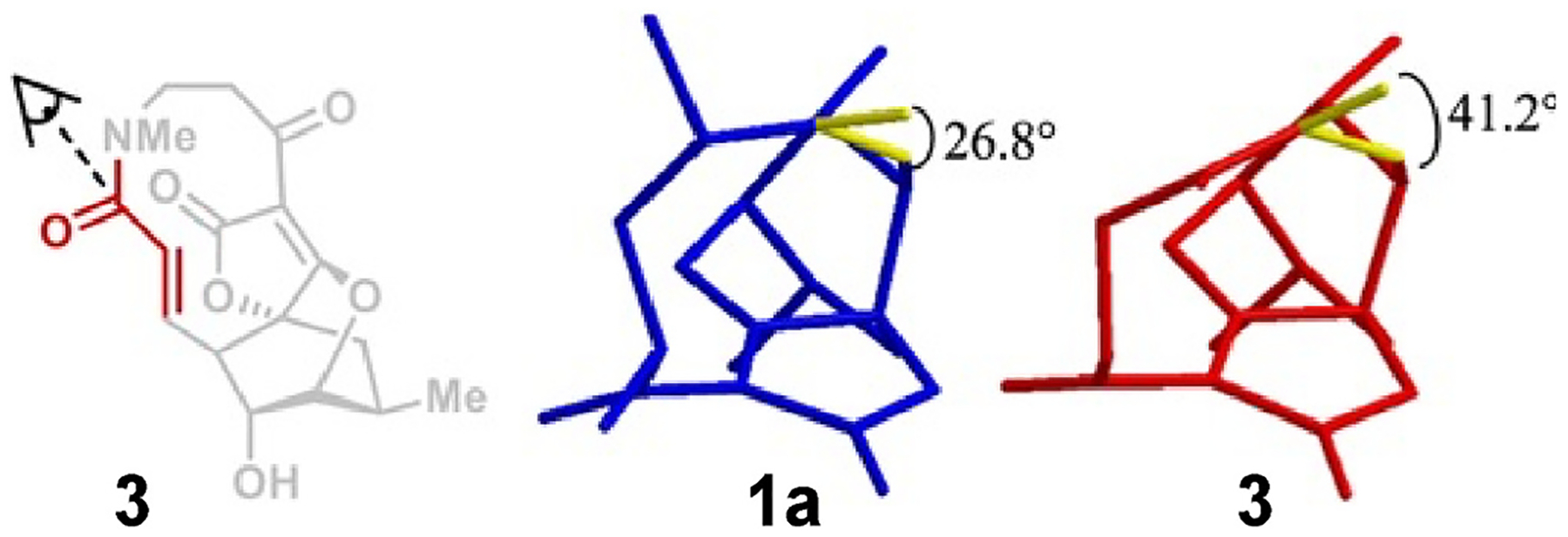
Conformational differences between the novel lactam analog **3** and atrop-abyssomicin C (**1a**). View of atrop-abyssomicin C (blue) and lactamabyssomicin (red) down the bond between the carbonyl and alkene (yellow) with respective angle measurements calculated from the crystal structure in Chem3D.

**Scheme 1.**
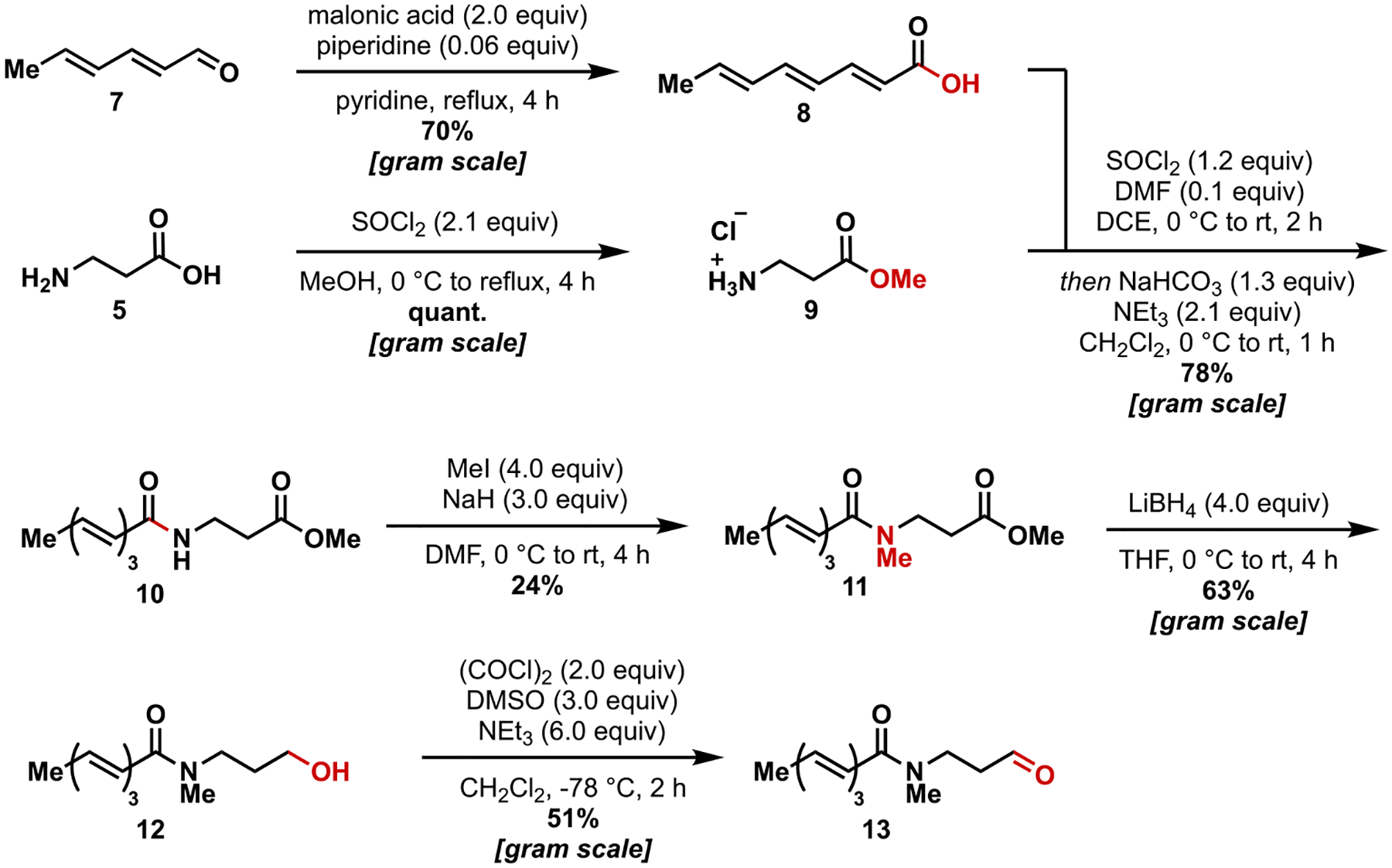
Synthesis of linear triene precursor.

**Scheme 2.**
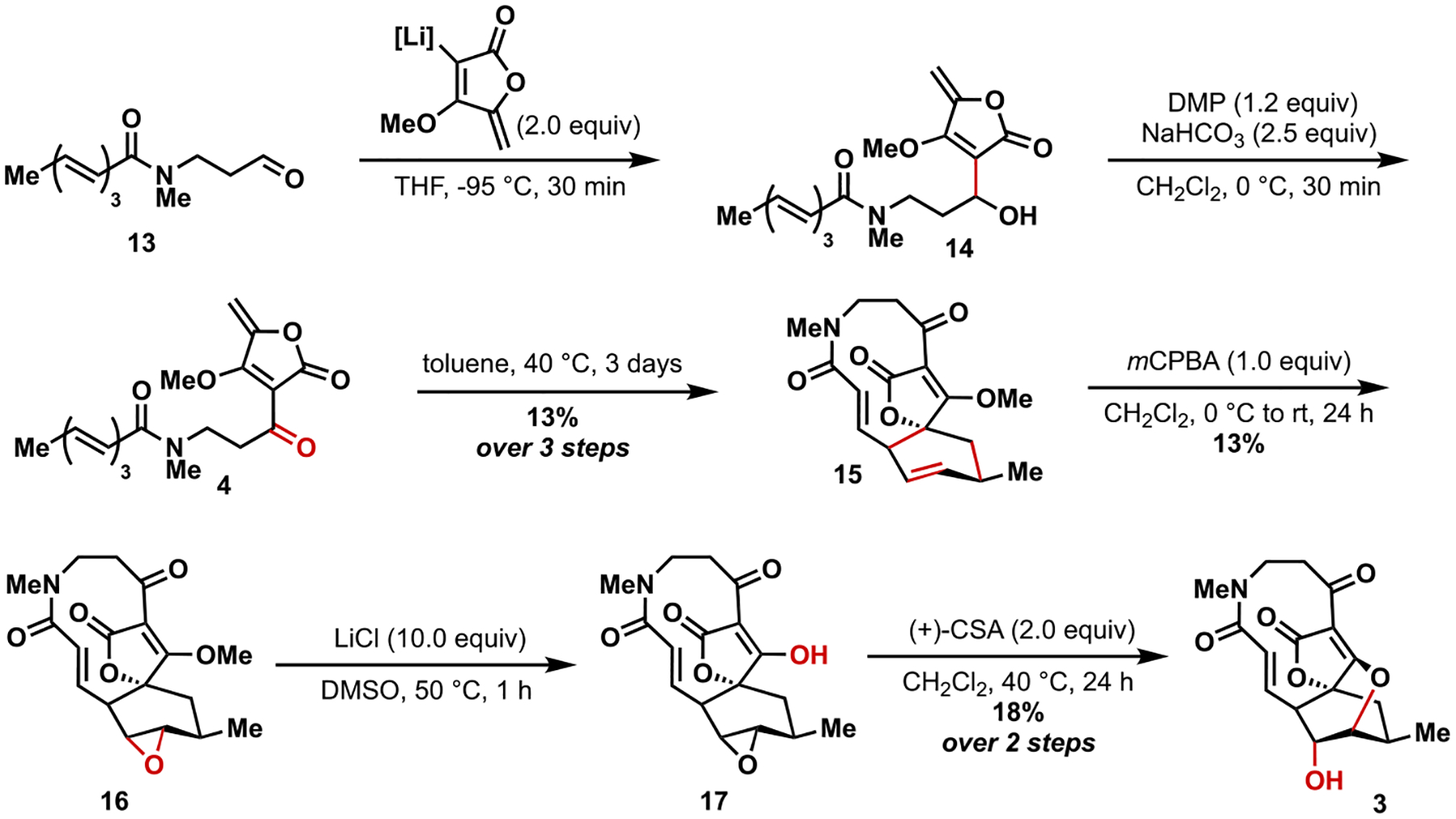
Completion of a lactam analog of abyssomicin C.

## Data Availability

Data will be made available on request.

## Appendix A. Supplementary data

Supplementary data to this article can be found online at https://doi.org/10.1016/j.tetlet.2025.155778.
