# Supplementary material for: Bioinspired synthesis of a lactam analogue of abyssomicin C
Source: Tetrahedron Lett. Author manuscript; Available in PMC 2026 Jun 2. (PMC13225853; doi:10.1016/j.tetlet.2025.155778)
Supplement: 1 [file NIHMS2180470-supplement-1.pdf]

Supporting Information

Bioinspired Synthesis of a Lactam Analog of Abyssomicin C

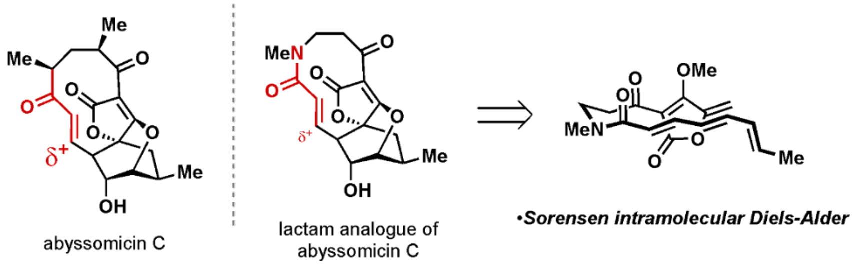

A. Cole Edwards[a] and Joshua G. Pierce*[a]

[a]Department of Chemistry, Comparative Medicine Institute and Integrative Sciences Initiative, North Carolina State University, Raleigh, NC 27607, USA

Table of Contents

Experimental……………………………………………………………………………..…S3

General Information…………………………………………………………………...S3

Synthetic Procedures and Spectra………………………………………………………..S4

Evaluation of Biological Activity…………………………………………………………S40

General Experimental…………………………………………………………………..S40

Broth microdilution method for determination of minimum inhibitory concentrations..S40

References…………………………………………………………………………………S41

Experimental

General Information

All chemicals were purchased from Fisher Scientific and used as received unless noted otherwise. Tetrahydrofuran and dichloromethane were dried using an alumina purification system. Toluene and diethyl ether were dried by stirring over 3Å molecular sieves for two days prior to use. Reactions were monitored by thin-layer chromatography (TLC) using pre-coated silica gel 60 F_{254} plates with 250 mm layer thickness. A 254 nm UV lamp was used for visualization as well as potassium permanganate (1.5 g of $KMnO_4$, 10 g of K_2CO_3, and 1.25 mL of 10% aqueous NaOH solution in 200 mL of water) cerium ammonium molybdate (0.2 g of $Ce_2(SO_4)_3$, 5.0 g of $(NH_4)_2MoO_4$, and 100 mL of 5% H_2SO_4) and anisaldehyde (5 mL of concentrated H_2SO_4, 1.5 mL of glacial acetic acid, and 3.7 mL of *p*-anisaldehyde in 135 mL of ethanol) stains. Alternatively, reactions could also be monitored by liquid-chromatography coupled with mass-spectrometry (LC-MS) using a Shimadzu LCMS-2020 equipped with a 2.6 mm C18 50 x 2.10 mm column. Column chromatography was carried out using a Biotage Isolera One accelerated chromatography instrument and linear solvent gradients were run on prepacked silica gel 20 or 60 μm columns unless manual chromatography was used in which 20-45 μm silica was used. ^{1}H NMR spectra were obtained on a 500, 600, or 700 MHz Bruker instrument in MeOD or $CDCl_3$ as indicated. Chemical shifts were reported in parts per million (ppm) with the residual solvent peak used as an internal standard. X-ray structure data was measured on a Bruker D8 VENTURE κ-geometry diffractometer system equipped with a Incoatec IµS 3.0 microfocus sealed tube (Cu Kα, λ = 1.54178 Å) and a multilayer mirror monochromator.

Synthetic Procedures and Spectra

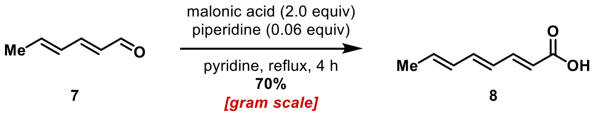

7 8

(2*E*,4*E*,6*E*)-Octa-2,4,6-trienoic acid (8) A 250 mL round-bottom flask was charged with a stir bar and flame-dried under vacuum, then cooled under argon. Pyridine (1.0 M, 83 ml) was added, followed by 2,4-hexadienal **7** (10.0 g, 83.2 mmol, 1.00 equiv), piperidine (0.43 g, 4.99 mmol, 0.06 equiv), and lastly malonic acid (17.5 g, 166 mmol, 2.00 equiv) and the solution was refluxed (115 °C) for 4 hours. After this time, complete consumption of the aldehyde was observed by TLC (visualized by UV and $KMnO_4$ stain) and the reaction flask was then cooled to 40 °C and poured over 166 mL of concentrated HCl on ice. This was then slowly swirled, the precipitate filtered off and the solid washed with ice cold water. The solid was dissolved in ethyl acetate and washed with a NaOH solution (1 M) three times. The aqueous layers were collected and reacidified to pH 2. This was then added to a separatory funnel and washed three times with ethyl acetate. The organic layers were then collected, washed with brine, dried (Na_2SO_4), and concentrated under reduced pressure. The resulting solid was redissolved in acetone and concentrated under reduced pressure to give 8.06 g (70%) of acid as a yellow solid: R_f = 0.83 (1:1 ethyl acetate: hexanes).

^{1}H NMR (500 MHz, $CDCl_3$) δ 7.38 (dd, *J* = 15.2, 11.4 Hz, 1H), 6.57 (dd, *J* = 14.9, 10.7 Hz, 1H), 6.23 (dd, *J* = 15.1, 11.5 Hz, 1H), 6.20 – 6.13 (m, 1H), 5.98 (dq, *J* = 14.1, 6.9 Hz, 1H), 5.84 (d, *J* = 15.2 Hz, 1H), 1.84 (d, *J* = 6.6 Hz, 1H).

^{13}C NMR (126 MHz, $CDCl_3$) δ 172.18, 147.40, 142.43, 136.20, 131.31, 127.46, 119.01, 18.78.

HRMS (*m/z*) $[M-H]^-$ calculated for $C_8H_9O_2$ 137.06080, found 137.06079.

IR (Diamond-ATR, neat) $\tilde{\nu}_{max}$: 2820, 1672, 1603, 1271, 1241, 997.

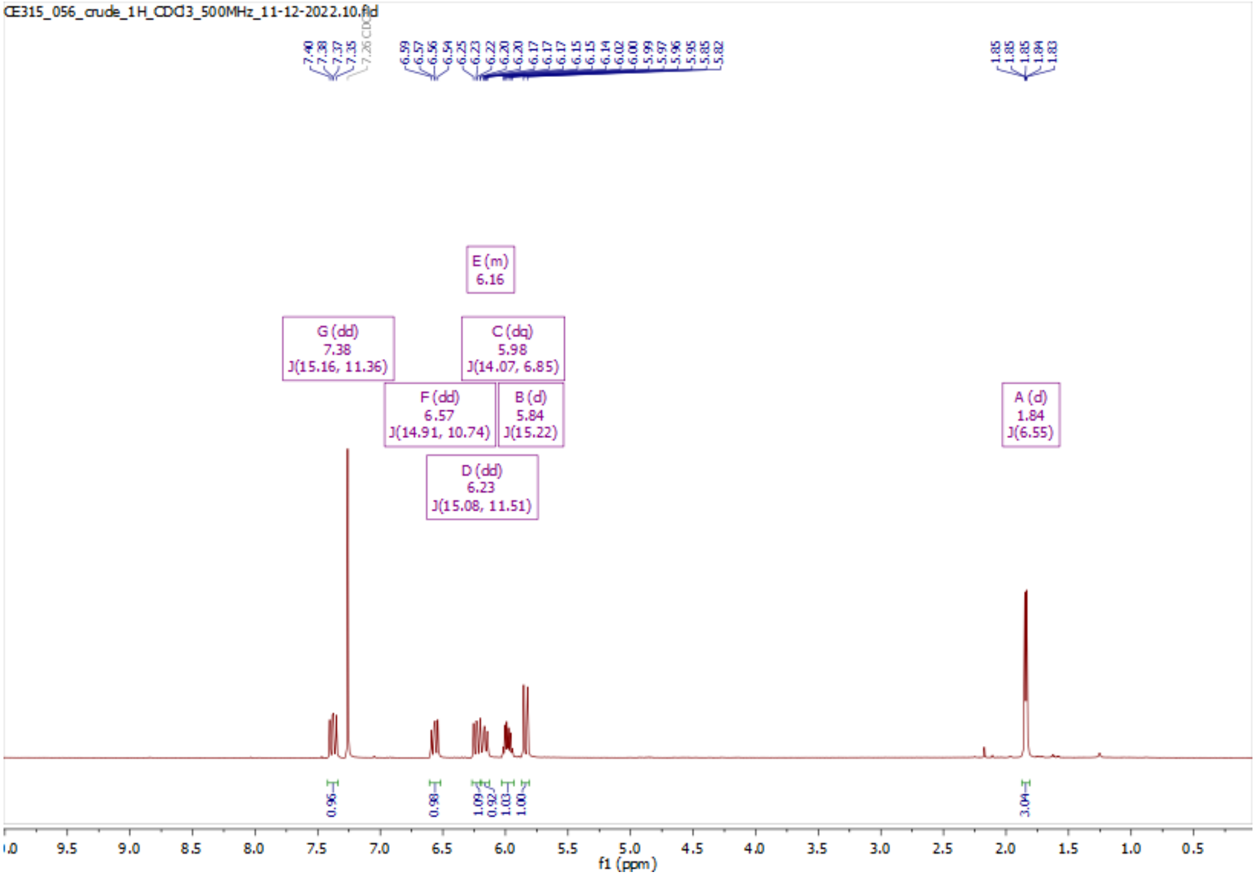
CE315_056_crude_1H_CDCl3_500MHz_11-12-2022.10.fid
G (dd)
7.38
J(15.16, 11.36)
E (m)
6.16
C (dq)
5.98
J(14.07, 6.85)
F (dd)
6.57
J(14.91, 10.74)
B (d)
5.84
J(15.22)
D (dd)
6.23
J(15.08, 11.51)
A (d)
1.84
J(6.55)
f1 (ppm)

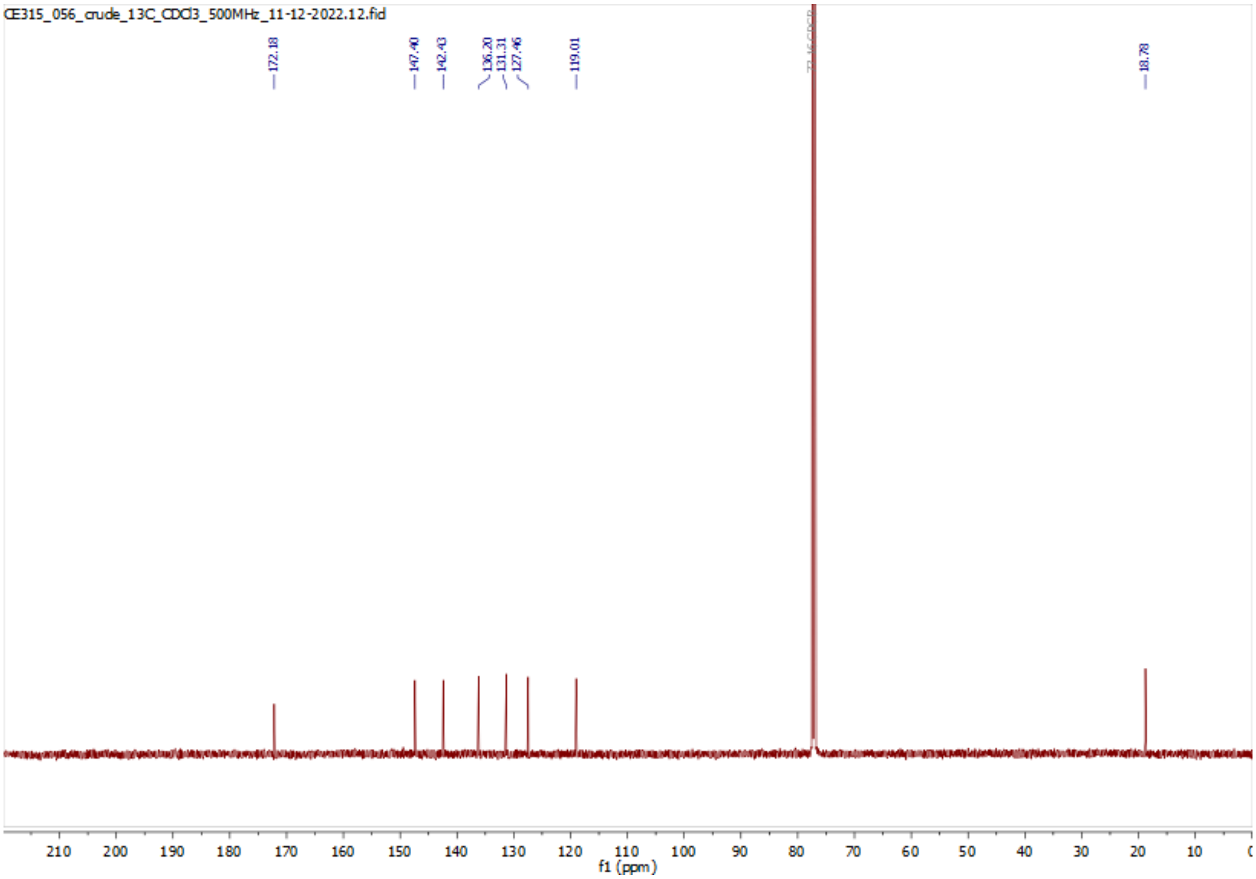
CE315_056_crude_13C_CDCl3_500MHz_11-12-2022.12.fid
172.18
147.40
142.43
136.20
131.31
127.46
119.01
18.78
210 200 190 180 170 160 150 140 130 120 110 100 90 80 70 60 50 40 30 20 10
f1 (ppm)

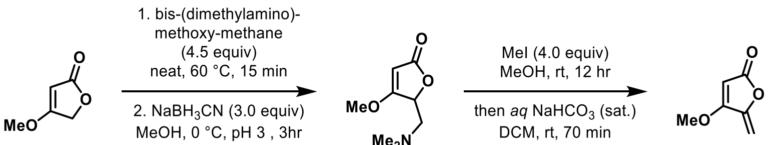

S1

4-Methoxy-5-methylenefuran-2(5H)-one (S1). Following the procedure found in the literature[1], a 50 mL round-bottom flask was charged with a stir bar, flame-dried under vacuum, and then cooled under an atmosphere of argon. 4-methoxy-5-methylenefuran-2(5H)-one (1.00 g, 8.50 mmol, 1.0 equiv) was added to the reaction vessel followed by bis-(dimethylamino)-methoxy-methane (5.32 g, 38.3 mmol, 4.5 equiv).* This was heated to 60 °C for 15 minutes, and then the unreacted bis-(dimethylamino)-methoxy-methane removed under reduced pressure. The residual oil was then dissolved in methanol (1.0 M, 8.5 mL) and the solution cooled to 0 °C in a water/ice bath. This was then treated with 4M methanolic hydrochloric acid solution (0.5 mL) resulting in an orange solution before portionwise addition of sodium cyanoborohydride (1.57 g, 25.5 mmol, 3 equiv). The reaction mixture was maintained acidic (pH 3, mixture is yellow at this pH) for 15 minutes by addition of 4M methanolic hydrochloric acid solution as required. After 3 hours, the methanol was removed under reduced pressure and the residual mixture extracted with ethyl acetate (3x) from an aqueous sodium hydroxide solution. The organic layers were combined, washed with brine, dried (Na_2SO_4), and concentrated under reduced pressure. The residual oil was redissolved in methanol (1.0 M, 8.5 mL) and the solution was treated with iodomethane (4.85 g, 34.0 mmol, 4.0 equiv). This was allowed to stir for 12 hours, and then the methanol was removed under reduced pressure. The residual oil was then redissolved in dichloromethane (1.0 M, 8.5 mL) and an aqueous, saturated sodium bicarbonate solution (17 mL) was added. This was allowed to stir for 70 minutes and then the two layers separated. The aqueous layer was then extracted with dichloromethane (3x), washed with brine, dried (Na_2SO_4), and concentrated under reduced pressure. The crude mixture was filtered through a silica plug with dichloromethane and then the filtrate concentrated under reduced pressure to yield 0.48 g (45%) of a white, needle-like solid. R_f = 0.30 (1:4 ethyl acetate: hexanes).

^{1}H NMR (500 MHz, $CDCl_3$) δ 5.25 (d, *J* = 0.9 Hz, 1H), 5.06 (dd, *J* = 2.7, 1.6 Hz, 1H), 5.03 (d, *J* = 2.2 Hz, 1H), 3.93 (s, 3H)

^{13}C NMR (126 MHz, $CDCl_3$) δ 169.83, 168.37, 149.81, 92.56, 90.09, 59.36.

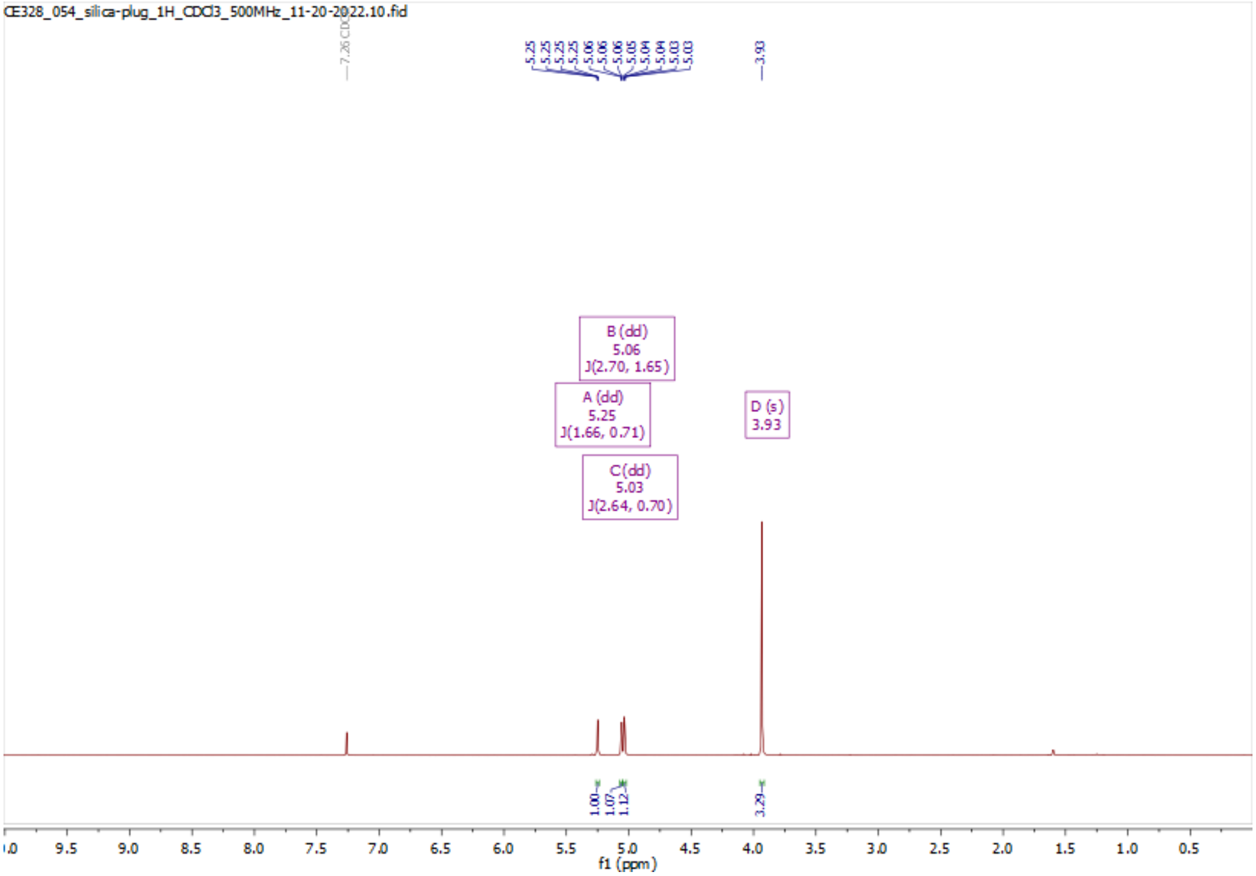

CE328_054_silica-plug_1H_CDCl3_500MHz_11-20-2022.10.fid
7.26 CDCl3
5.25
5.25
5.25
5.25
5.06
5.06
5.06
5.05
5.04
5.04
5.03
5.03
3.93
B (dd)
5.06
J(2.70, 1.65)
A (dd)
5.25
J(1.66, 0.71)
D (s)
3.93
C (dd)
5.03
J(2.64, 0.70)
1.00
1.07
1.12
3.29
9.5
9.0
8.5
8.0
7.5
7.0
6.5
6.0
5.5
5.0
4.5
4.0
3.5
3.0
2.5
2.0
1.5
1.0
0.5
f1 (ppm)

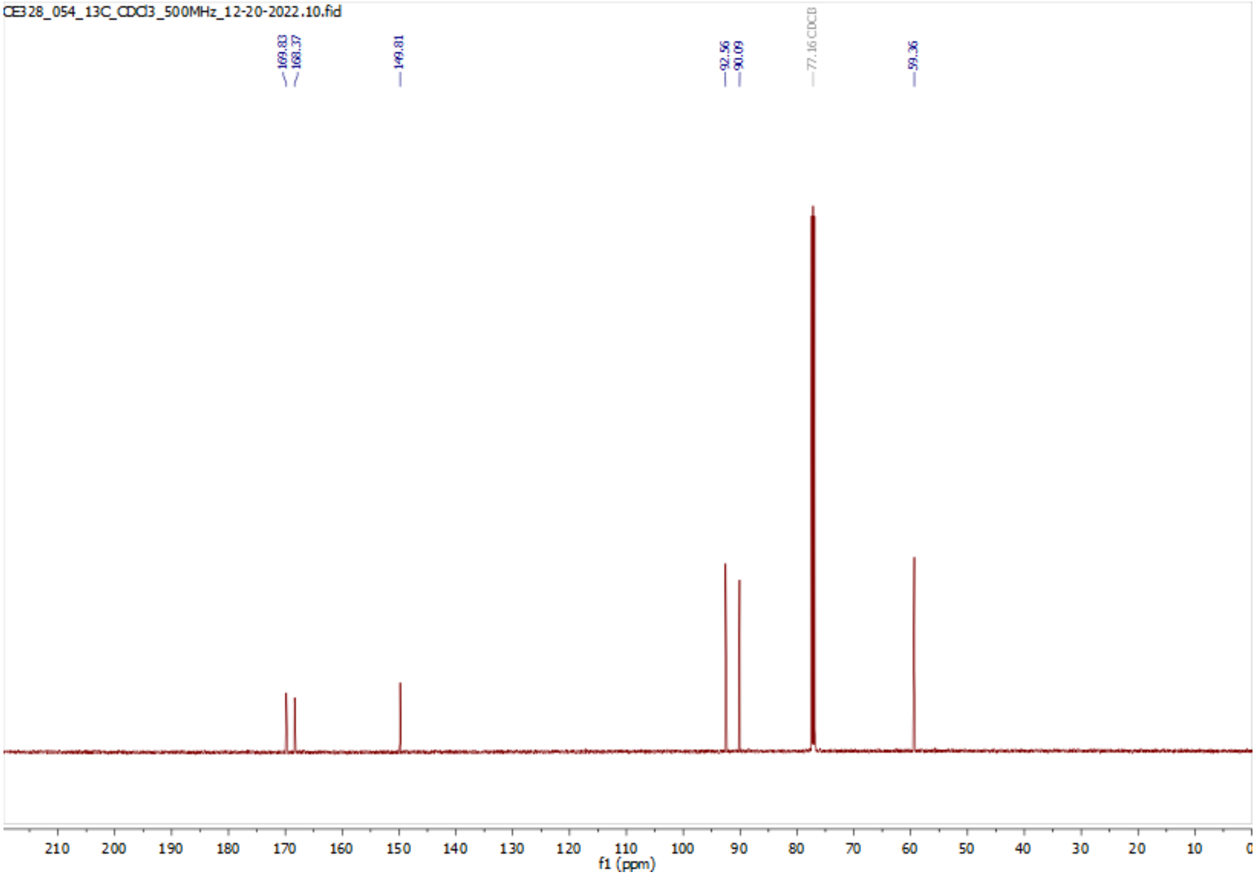

*During the time of this synthesis, the bis-(dimethylamino)-methoxy-methane was discontinued from the supplier, so this was replaced with N,N-dimethylformamide dimethyl acetal which required heating to 100 °C for 4 hours.

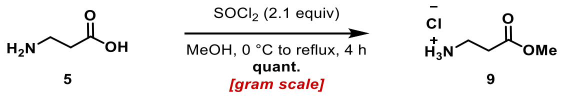

Methyl 3-aminopropanoate hydrochloride (9) Following the procedure found in the literature[2], a 500 mL round-bottom flask was charged with a stir bar, flame-dried under vacuum, and cooled under an atmosphere of argon. β-alanine **5** (10.0 g, 111.1 mmol, 1.00 equiv) was added followed by methanol (1.1 M, 100 mL) and the reaction flask cooled to 0 °C. After 5 minutes, thionyl chloride (18.7 g, 155.6 mmol, 1.4 equiv) was added in a rapid dropwise fashion and after complete addition the ice bath was taken away and the reaction flask warmed to room temperature. Then the reaction flask was heated to reflux (64 °C) for 4 hours. At the end of this time, the methanol was removed by evaporation and the residual solid filtered off with diethyl ether to give 31.3 g (quant.) of a white solid used without further purification.

^{1}H NMR (500 MHz, MeOD) δ 3.75 (s, 3H), 3.22 (t, *J* = 6.6 Hz, 2H), 2.76 (t, *J* = 6.5 Hz, 2H).

^{13}C NMR (126 MHz, MeOD) δ 172.55, 52.64, 36.44, 32.05.

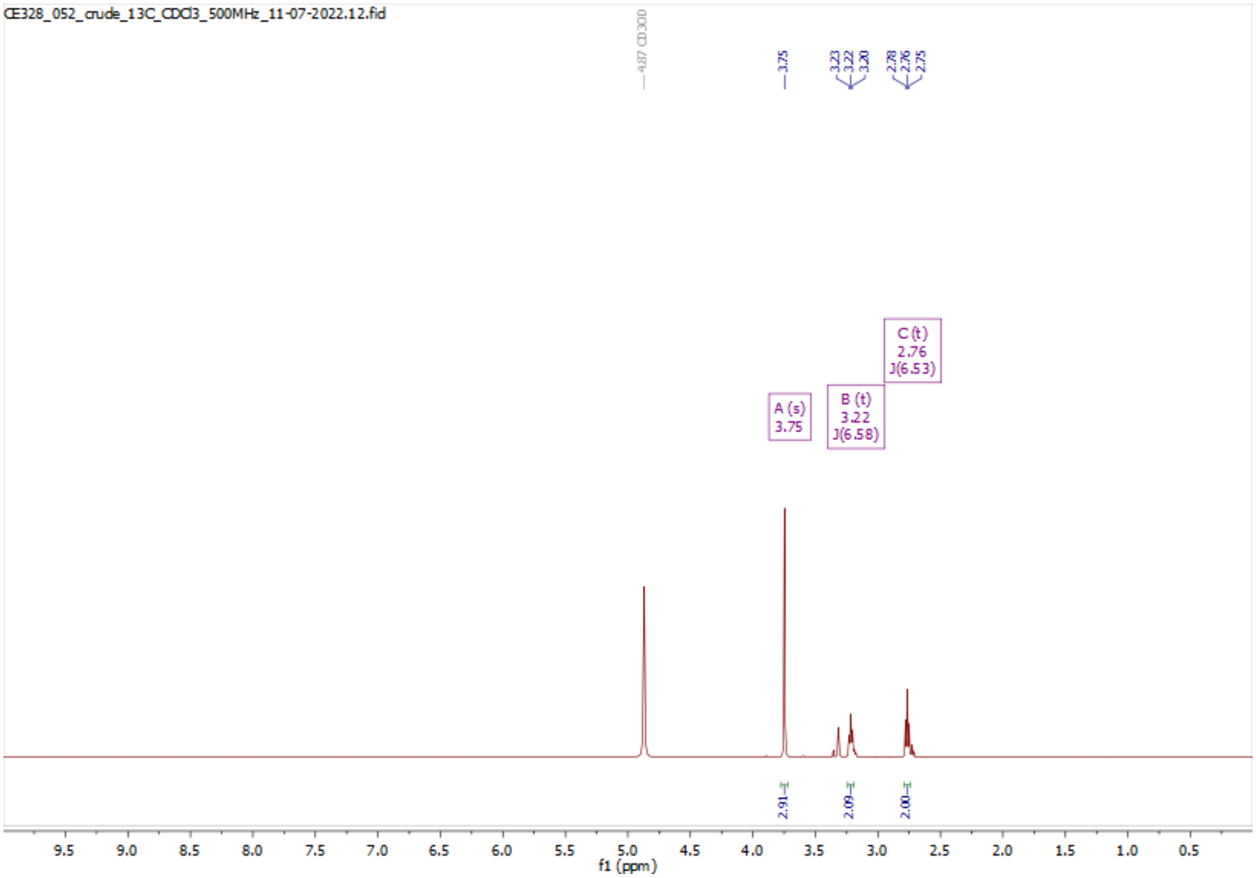

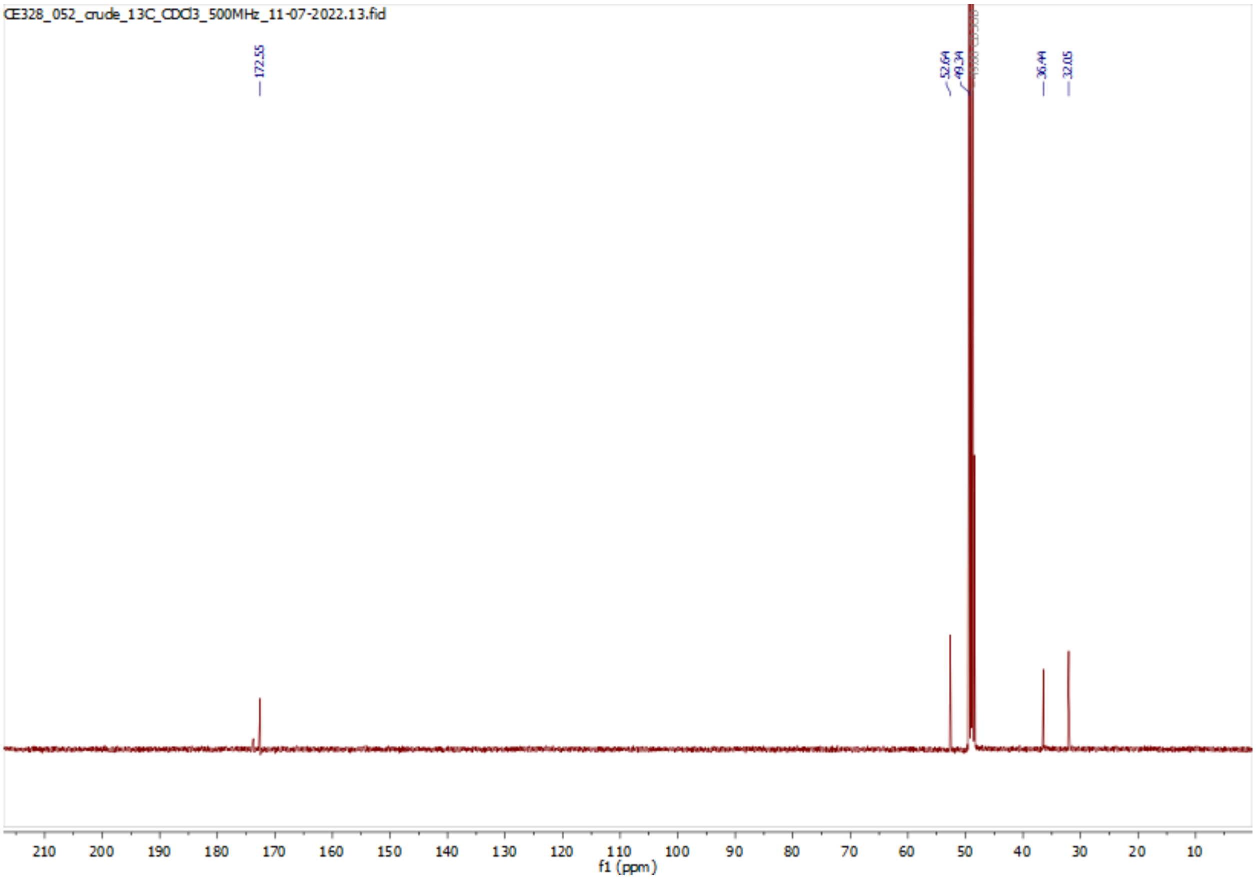

CE328_052_crude_13C_CDCl3_500MHz_11-07-2022.13.fid
172.55
52.64
49.34
36.44
32.05
210
200
190
180
170
160
150
140
130
120
110
100
90
80
70
60
50
40
30
20
10
f1 (ppm)

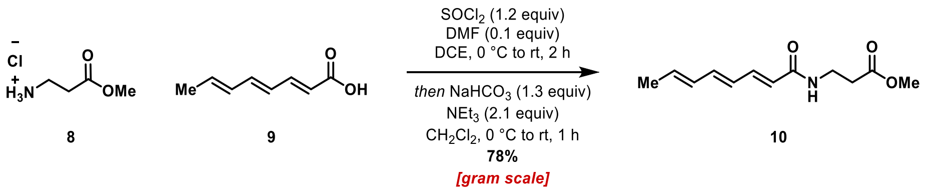

Methyl 3-((2E,4E,6E)-octa-2,4,6-trienamido)propanoate (10) A 250 mL round-bottom flask was charged with a stir bar, flame-dried under vacuum, and then cooled under an atmosphere of argon. (2*E*,4*E*,6*E*)-octa-2,4,6-trienoic acid **9** (4.16 g, 30.1 mmol, 1.0 equiv) was added to the reaction vessel followed by dichloroethane (0.5 M, 60.0 mL). Then N,N-dimethylformamide (0.22 g, 3.01 mmol, 0.1 equiv) was added and the reaction vessel cooled to 0 °C in a water/ice bath. This was stirred for 5 minutes and then thionyl chloride (4.29 g, 36.1 mmol, 1.2 equiv) was added dropwise. This was allowed to stir for 2 hours until a solution was made. Then the reaction was concentrated under reduced pressure and the resulting oil put on high vac. Next a 500 mL round-bottom flask was charged with a stir bar, flame dried under vacuum, and then cooled under an atmosphere of argon. The total volume of dichloromethane used in this step was 100.3 mL (0.3 M). Three-quarters of this volume (75 mL) was added to this new flask, followed by methyl 3-aminopropanoate hydrochloride **8** (8.40 g, 60.2 mmol, 2.0 equiv). Then triethylamine (6.46 g, 63.2 mmol, 2.1 equiv) was added dropwise, followed by sodium bicarbonate (3.03 g, 36.1 mmol, 1.2 equiv). This flask was then put into a water/ice bath to cool while the next solution was made. The acid chloride was put under an atmosphere of argon and taken off high vac. Then a solution was made using the other equivalent of dichloromethane (25 mL). This solution was added to the solution of methyl 3-aminopropanoate and the reaction allowed to stir for 1 hour. Then the reaction was diluted with dichloromethane and then quenched with water (50 mL). The organic and aqueous layers were separated, and the aqueous layer extracted with dichloromethane (3x). The organic layers were collected, washed with brine, dried (Na_2SO_4), and then concentrated under reduced pressure. The resulting crude material (4.92 g, 78%) was a brown solid and was used without further purification.

^{1}H NMR (500 MHz, $CDCl_3$) δ 7.21 (dd, *J* = 14.9, 11.3 Hz, 1H), 6.48 (dd, *J* = 14.9, 10.7 Hz, 1H), 6.19 – 6.07 (m, 2H), 5.89 (dq, *J* = 14.1, 6.8 Hz, 1H), 5.78 (d, *J* = 15.0 Hz, 1H), 3.69 (s, 3H), 3.59 (q, *J* = 6.0 Hz, 2H), 2.58 (t, *J* = 5.9 Hz, 2H), 1.80 (d, *J* = 6.6 Hz, 3H).

^{13}C NMR (126 MHz, $CDCl_3$) δ 173.37, 166.27, 141.48, 140.14, 134.31, 131.40, 127.69, 122.57, 51.94, 34.98, 33.98, 18.65.

HRMS (*m*/*z*) $[M+H]^+$ calculated for $C_{12}H_{18}NO_3$ 224.12812, found 224.12813, $[M+Na]^+$ calculated for $C_{12}H_{17}NO_3Na$ 246.11006, found 246.11003.

IR (Diamond-ATR, neat) $\tilde{\nu}_{max}$: 3280, 2950, 1728, 1605, 1539, 1172, 1154, 1006.

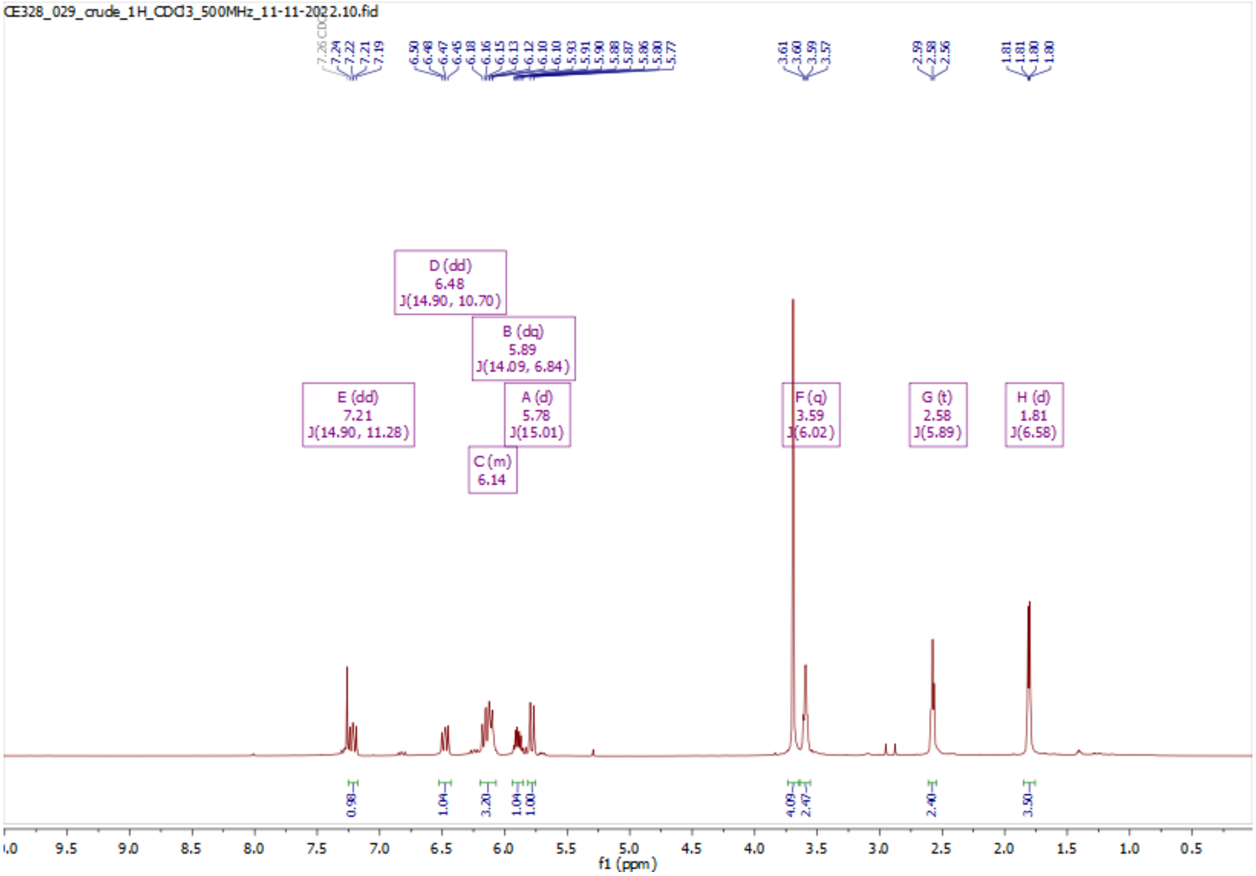
CE328_029_crude_1H_CDCl3_500MHz_11-11-2022.10.fid
7.26 CDCl3
7.24
7.22
7.21
7.19
6.50
6.48
6.47
6.45
6.18
6.16
6.15
6.13
6.12
6.10
6.10
5.93
5.91
5.90
5.88
5.87
5.86
5.80
5.77
3.61
3.60
3.59
3.57
2.59
2.58
2.56
1.81
1.81
1.80
1.80
D (dd) 6.48 J(14.90, 10.70)
B (dq) 5.89 J(14.09, 6.84)
E (dd) 7.21 J(14.90, 11.28)
A (d) 5.78 J(15.01)
C (m) 6.14
F (q) 3.59 J(6.02)
G (t) 2.58 J(5.89)
H (d) 1.81 J(6.58)
0.98
1.04
3.20
1.04
1.00
4.09
2.47
2.40
3.50
9.5 9.0 8.5 8.0 7.5 7.0 6.5 6.0 5.5 5.0 4.5 4.0 3.5 3.0 2.5 2.0 1.5 1.0 0.5
f1 (ppm)

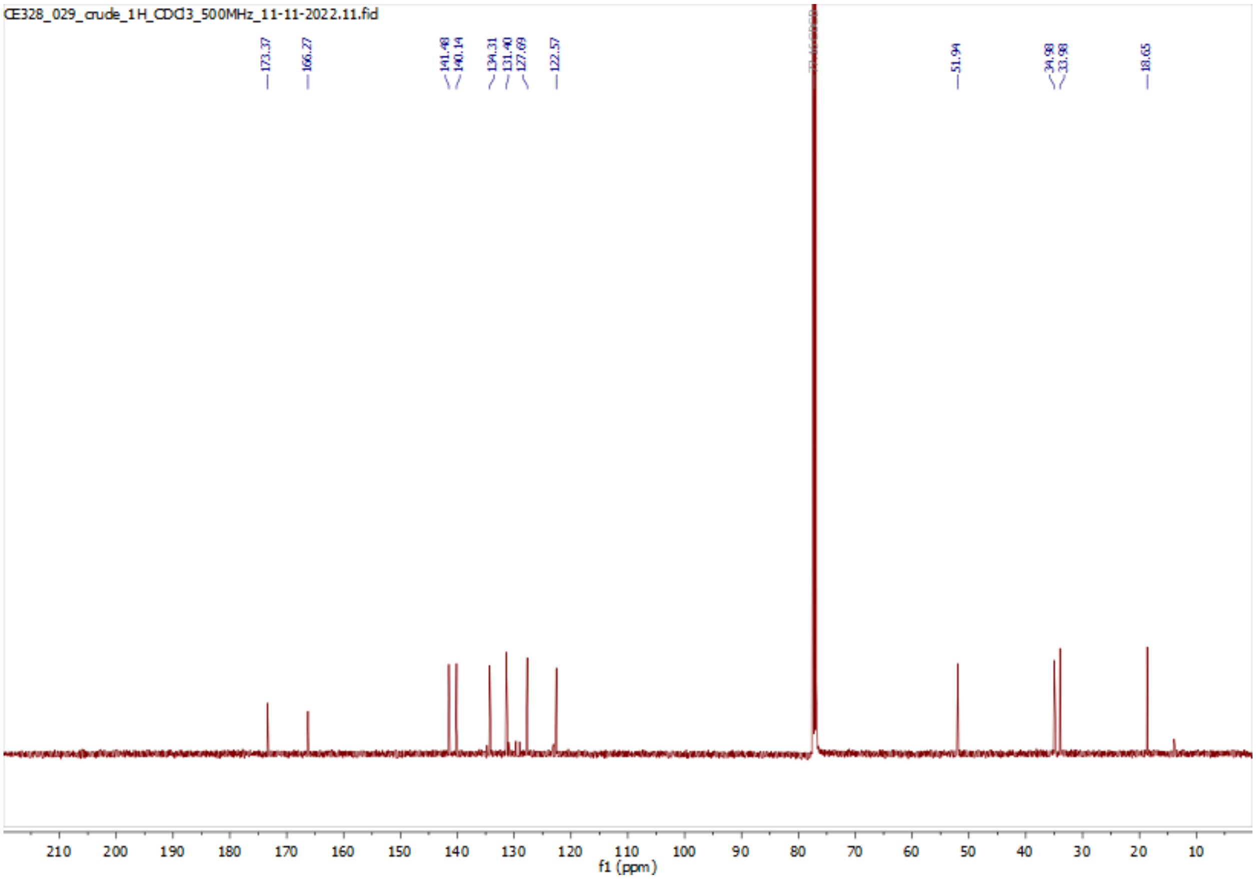
CE328_029_crude_1H_CDCl3_500MHz_11-11-2022.11.fid
173.37
166.27
141.48
140.14
134.31
131.40
127.69
122.57
51.94
34.98
33.98
18.65
210
200
190
180
170
160
150
140
130
120
110
100
90
80
70
60
50
40
30
20
10
f1 (ppm)

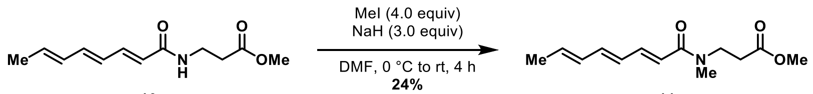

Methyl 3-((2E,4E,6E)-N-methylocta-2,4,6-trienamido)propanoate (11) A 100 mL round-bottom flask was charged with a stir bar, flame-dried under vacuum, and then cooled under an atmosphere of argon. A suspension of sodium hydride (0.13 g, 3.4 mmol, 1.5 equiv) was made in *N*,*N*-dimethylformamide (0.2 M, 11 mL) and the reaction vessel cooled to 0 °C. Then methyl 3-((2E,4E,6E)-octa-2,4,6-trienamido)propanoate **10** (0.50 g, 2.24 mmol, 1.0 equiv) was added to the reaction vessel in one portion and after 5 minutes iodomethane (0.64 g, 4.48 mmol, 2.0 equiv) was added. After 2 hours, a second portion of sodium hydride (0.13 g, 3.4 mmol, 1.5 equiv) and iodomethane (0.64 g, 4.48 mmol, 2.0 equiv) were added. After 4 hours the reaction was diluted with diethyl ether (20 mL) and quenched with a saturated, aqueous ammonium chloride solution (10 mL). Then the organic layer was separated, and the aqueous layer was extracted with diethyl ether (3x). Then the organic layers were collected, washed with water (2x), then washed with brine, dried (Na_2SO_4), and concentrated under reduced pressure. The resulting oil still had grease present from the sodium hydride, so this was removed using hexanes, and the resulting dark oil was purified using automated chromatography. This resulted in 0.127 g of a yellow oil (24%) that was a mixture of two inseparable, rotational isomers. R_f = 0.35 (60% ethyl acetate in hexanes) UV active, stains with $KMnO_4$ and anisaldehyde.

^{1}H NMR (500 MHz, $CDCl_3$) δ 7.29 (dd, *J* = 14.7, 11.3 Hz, 1H), 6.48 (dd, *J* = 14.9, 10.7 Hz, 1H), 6.30 – 6.18 (m, 2H), 6.12 (ddd, *J* = 15.0, 10.7, 1.8 Hz, 1H), 5.89 (dq, *J* = 14.0, 6.8 Hz, 1H), 3.72 – 3.64 (m, 5H), 3.16 – 2.98 (m, 3H), 2.60 (t, *J* = 7.0 Hz, 2H), 1.80 (dd, *J* = 6.9, 1.7 Hz, 3H).

^{13}C NMR (176 MHz, $CDCl_3$) δ 173.31, 172.72, 171.59, 166.90, 143.46, 143.16, 140.08, 134.71, 134.25, 131.44, 130.92, 129.12, 128.23, 122.64, 119.32, 118.82, 52.07, 51.83, 45.74, 44.96, 36.53, 34.07, 33.81, 32.60, 18.63, 13.89.

HRMS (*m/z*) $[M+H]^+$ calculated for $C_{13}H_{20}O_3N$ 238.14377, found 238.14363, $[M+Na]^+$ calculated for $C_{13}H_{19}O_3NNa$ 260.12571, found 260.12561, $[2M+Na]^+$ calculated for $C_{26}H_{38}O_6N_2Na$ 497.26221, found 497.26215.

IR (Diamond-ATR, neat) $\tilde{\nu}_{max}$: 2946, 1728, 1601, 1005.

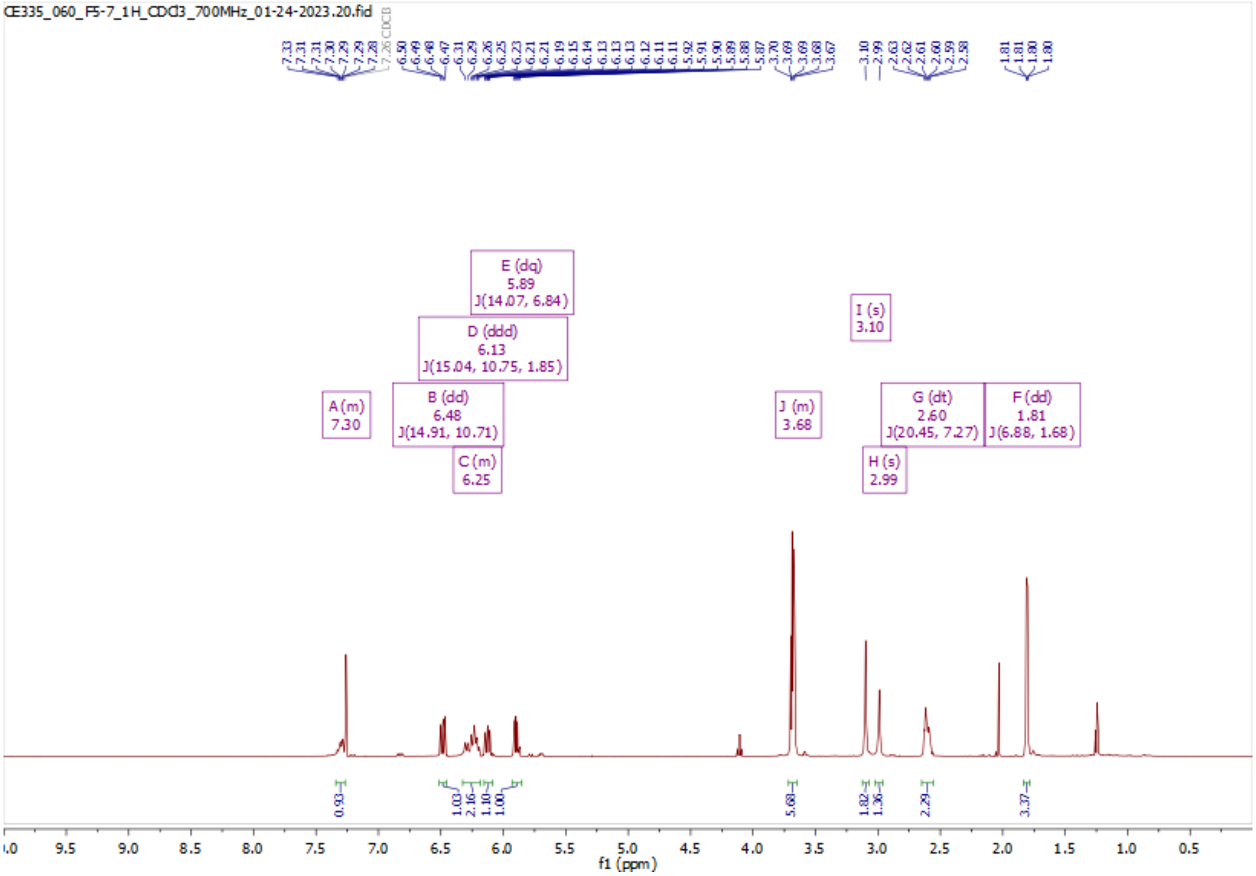
CE335_060_F5-7_1H_CDCl3_700MHz_01-24-2023.20.fid
A (m)
7.30
B (dd)
6.48
J(14.91, 10.71)
C (m)
6.25
D (ddd)
6.13
J(15.04, 10.75, 1.85)
E (dq)
5.89
J(14.07, 6.84)
J (m)
3.68
I (s)
3.10
H (s)
2.99
G (dt)
2.60
J(20.45, 7.27)
F (dd)
1.81
J(6.88, 1.68)
f1 (ppm)

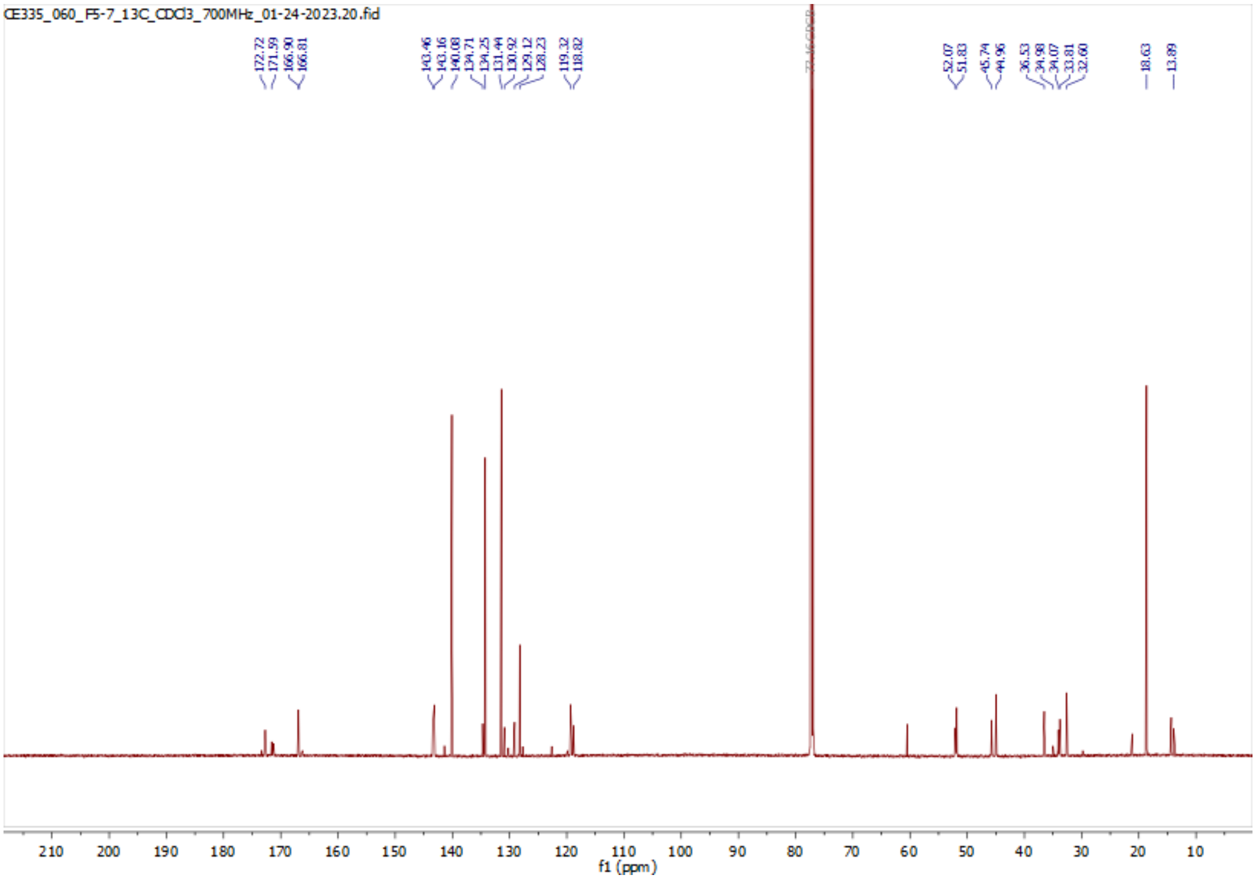

CE335_060_F5-7_13C_CDCl3_700MHz_01-24-2023.20.fid
172.72
171.59
166.90
166.81
143.46
143.16
140.08
134.71
134.25
131.44
130.92
129.12
128.23
119.32
118.82
77.16 CDCl3
52.07
51.83
45.74
44.96
36.53
34.98
34.07
33.81
32.60
18.63
13.89
f1 (ppm)

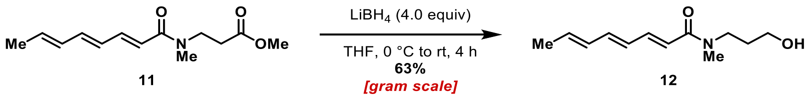

(2E,4E,6E)-*N*-(3-Hydroxypropyl)-*N*-methylocta-2,4,6-trienamide (12) A 100 mL round-bottom flask was charged with a stir bar, flame-dried under vacuum, and cooled under an atmosphere of argon. A suspension of lithium borohydride (0.87 g, 37.8 mmol, 4.0 equiv) was made in tetrahydrofuran (0.3 M, 28 mL) and the reaction vessel cooled to 0 °C. After a minute, methyl 3-((2E,4E,6E)-N-methylocta-2,4,6-trienamido)propanoate **11** (2.24 g, 9.46 mmol, 1.0 equiv) was added as a solution in tetrahydrofuran (4 mL). After 2 hours the reaction was cooled to 0 °C, diluted with ethyl acetate (20 mL), and quenched by dropwise addition of a saturated, aqueous ammonium chloride solution (10 mL). The organic and aqueous layers were separated, and the aqueous layer extracted with ethyl acetate (3x). The organic layers were collected, washed with brine, dried (Na_2SO_4), and concentrated under reduced pressure. Automated chromatography was used to separate the product from a more non-polar product to yield 1.26 g (63%) as a yellow oil that was a mixture of two inseparable, rotational isomers. R_f = 0.33 (100% ethyl acetate) This was stored in benzene at -30 °C until the next step.

^{1}H NMR (500 MHz, $CDCl_3$) δ 7.33 (dd, *J* = 14.6, 11.3 Hz, 1H), 6.51 (dd, *J* = 14.9, 10.7 Hz, 1H), 6.28 (d, *J* = 15.6 Hz, 1H), 6.23 (dd, *J* = 15.0, 11.4 Hz, 1H), 6.14 (dd, *J* = 15.2, 10.8 Hz, 1H), 3.59 (t, *J* = 6.0 Hz, 2H), 3.48 (t, *J* = 5.5 Hz, 2H), 3.05 (s, 3H), 1.82 (d, *J* = 6.9 Hz, 4H), 1.72 (d, *J* = 6.2 Hz, 2H).

^{13}C NMR (126 MHz, $CDCl_3$) δ 168.20, 144.10, 140.67, 134.76, 131.39, 128.03, 118.43, 57.88, 44.34, 35.41, 29.65, 18.68.

HRMS (*m*/*z*) $[M+H]^+$ calculated for $C_{12}H_{20}O_2N$ 210.14886, found 210.14871, $[M+Na]^+$ calculated for $C_{12}H_{19}O_2NNa$ 232.13080, found 232.13067, $[2M+Na]^+$ calculated for $C_{24}H_{38}O_4N_2Na$ 441.27238, found 441.27227.

IR (Diamond-ATR, neat) $\tilde{v}_{max}$: 3373, 2928, 2874, 1735, 1625, 1579, 1398, 1057, 1001.

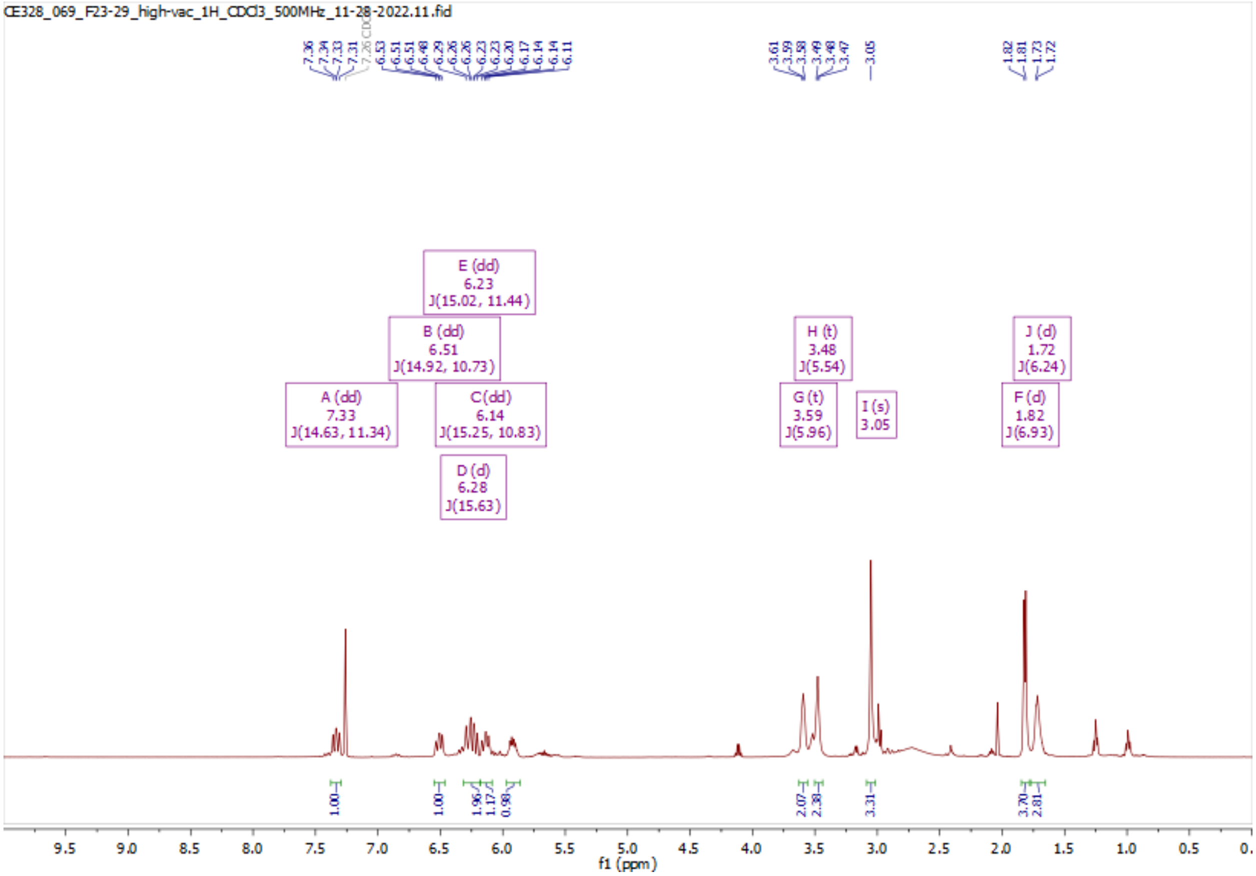

CE328_069_F23-29_high-vac_1H_CDCl3_500MHz_11-28-2022.11.fid
A (dd) 7.33 J(14.63, 11.34)
B (dd) 6.51 J(14.92, 10.73)
C (dd) 6.14 J(15.25, 10.83)
D (d) 6.28 J(15.63)
E (dd) 6.23 J(15.02, 11.44)
F (d) 1.82 J(6.93)
G (t) 3.59 J(5.96)
H (t) 3.48 J(5.54)
I (s) 3.05
J (d) 1.72 J(6.24)
f1 (ppm)

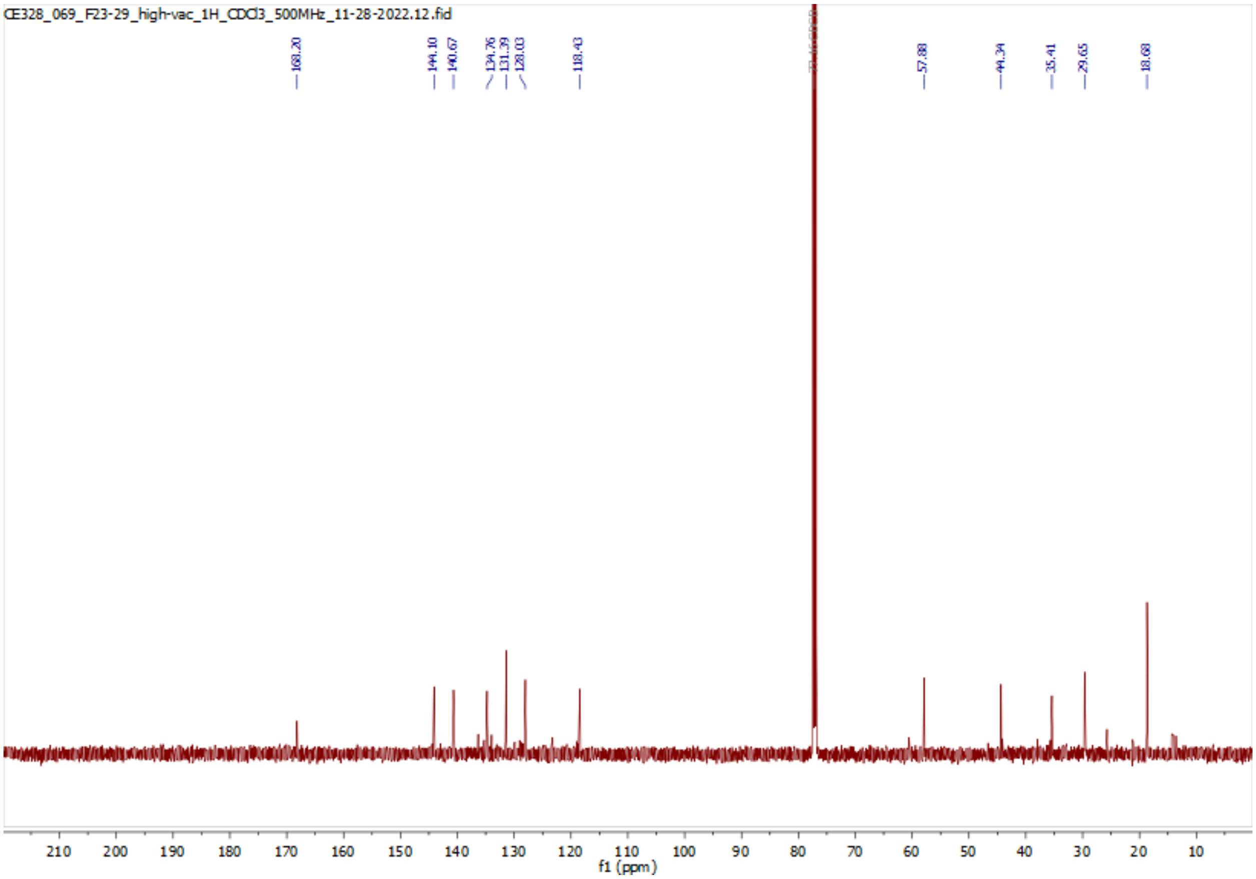

CE328_069_F23-29_high-vac_1H_CDCl3_500MHz_11-28-2022.12.fid
168.20
144.10
140.67
134.76
131.39
128.03
118.43
57.88
44.34
35.41
29.65
18.68
210
200
190
180
170
160
150
140
130
120
110
100
90
80
70
60
50
40
30
20
10
f1 (ppm)

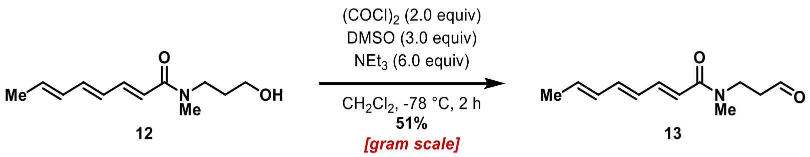

(2E,4E,6E)-*N*-Methyl-N-(3-oxopropyl)octa-2,4,6-trienamide (13) A 250 mL round-bottom flask was charged with a stir bar, flame-dried under vacuum, and then cooled under an atmosphere of argon. A solution of oxalyl chloride (1.55 g, 12.0 mmol, 2.0 equiv) was made in dichloromethane (0.1 M, 48 mL added here). The reaction vessel was then cooled to -78 °C and allowed to stir for 5 minutes. A second solution was made of dimethylsulfoxide (1.41 g, 18.0 mmol, 3.0 equiv) in dichloromethane (4.0 mL) and this was added to the solution of oxalyl chloride dropwise, then allowed to stir for 30 minutes. After this time had passed, a solution of (2E,4E,6E)-N-(3-hydroxypropyl)-N-methylocta-2,4,6-trienamide **12** (1.26 g, 6.00 mmol, 1.0 equiv) in dichloromethane (8.0 mL) was added to the reaction flask in a dropwise manner, and the reaction allowed to stir for 1 hour. Then the triethylamine (3.68 g, 36.0 mmol, 6 equiv) was added dropwise and after 30 minutes then reaction flask allowed to warm up to room temperature. Then the reaction was diluted with dichloromethane and quenched with water (60 mL). The organic and aqueous layers were then separated, and the aqueous layer extracted with dichloromethane (3x). The organic layers were collected, washed with an aqueous 1% HCl solution (40 mL), followed by an aqueous 5% sodium carbonate solution (40 mL). Then the organic layer was washed with water, dried (Na_2SO_4), and concentrated under reduced pressure and purified using automated chromatography to yield 0.64 g (51%) of a yellow solid that was a mixture of two inseparable, isomers. R_f = 0.42 (4:1 ethyl acetate: hexanes).

^{1}H NMR (500 MHz, $CDCl_3$) δ 9.79 (s, 1H), 7.27 (q, *J* = 9.6, 7.7 Hz, 1H), 6.48 (dd, *J* = 14.9, 10.7 Hz, 1H), 6.28 – 6.17 (m, 2H), 6.12 (ddd, *J* = 15.0, 10.7, 1.8 Hz, 1H), 5.89 (dq, *J* = 14.1, 6.9 Hz, 1H), 3.71 (t, *J* = 6.6 Hz, 2H), 3.09 (s, 3H), 2.79 – 2.72 (m, 2H), 1.80 (dd, *J* = 7.0, 1.6 Hz, 3H)

^{13}C NMR (126 MHz, $CDCl_3$) δ 201.09, 167.06, 143.37, 140.27, 134.43, 131.41, 128.14, 119.06, 42.76, 42.60, 36.48, 18.66.

HRMS (*m*/*z*) $[M+H]^+$ calculated for $C_{12}H_{18}O_2N$ 208.13321, found 208.13317, $[M+Na]^+$ calculated for $C_{12}H_{17}O_2NNa$ 230.11515, found 230.11512.

IR (Diamond-ATR, neat) $\tilde{v}_{max}$: 2924, 2850, 2727, 1716, 1629, 1582.

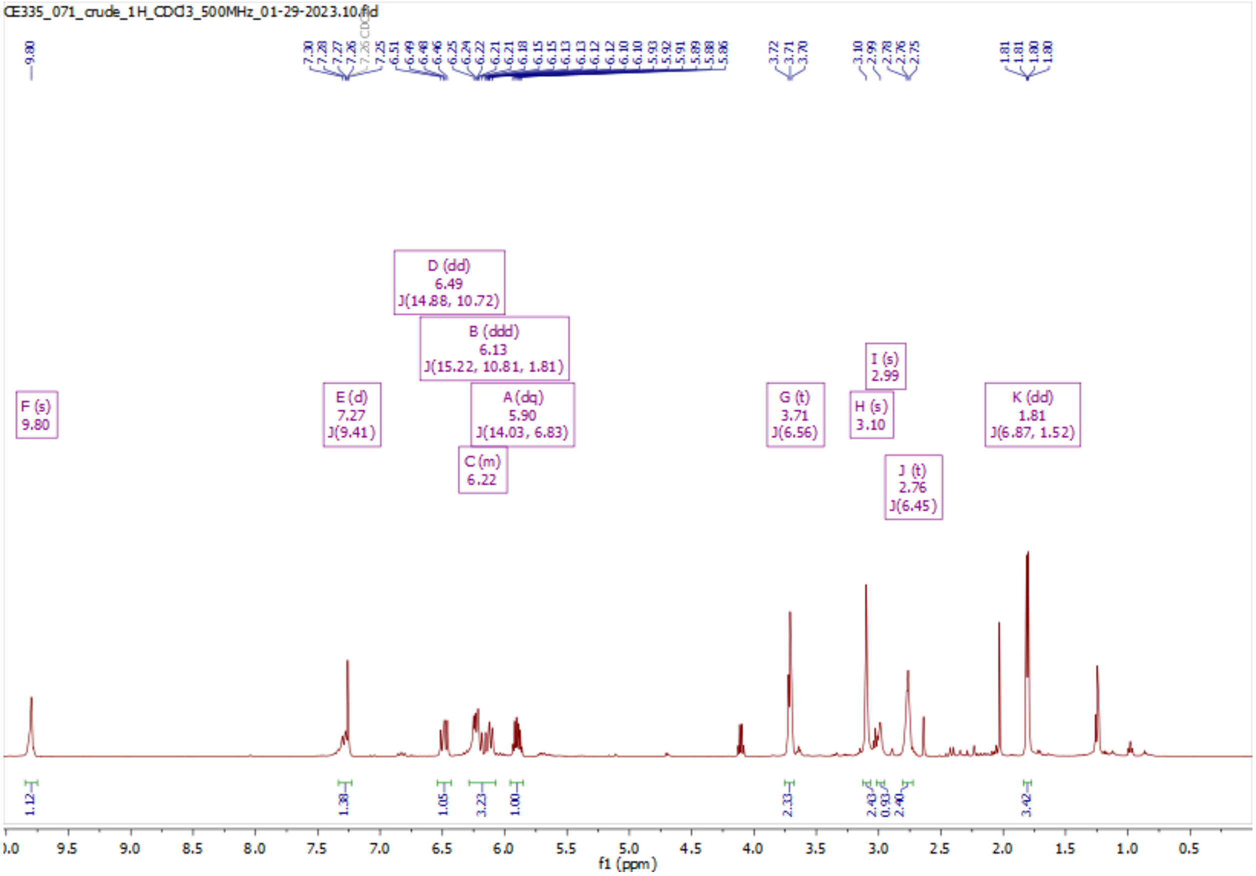
CE335_071_crude_1H_CDCl3_500MHz_01-29-2023.10.fid
D (dd)
6.49
J(14.88, 10.72)
B (ddd)
6.13
J(15.22, 10.81, 1.81)
F (s)
9.80
E (d)
7.27
J(9.41)
A (dq)
5.90
J(14.03, 6.83)
C (m)
6.22
G (t)
3.71
J(6.56)
I (s)
2.99
H (s)
3.10
J (t)
2.76
J(6.45)
K (dd)
1.81
J(6.87, 1.52)
f1 (ppm)

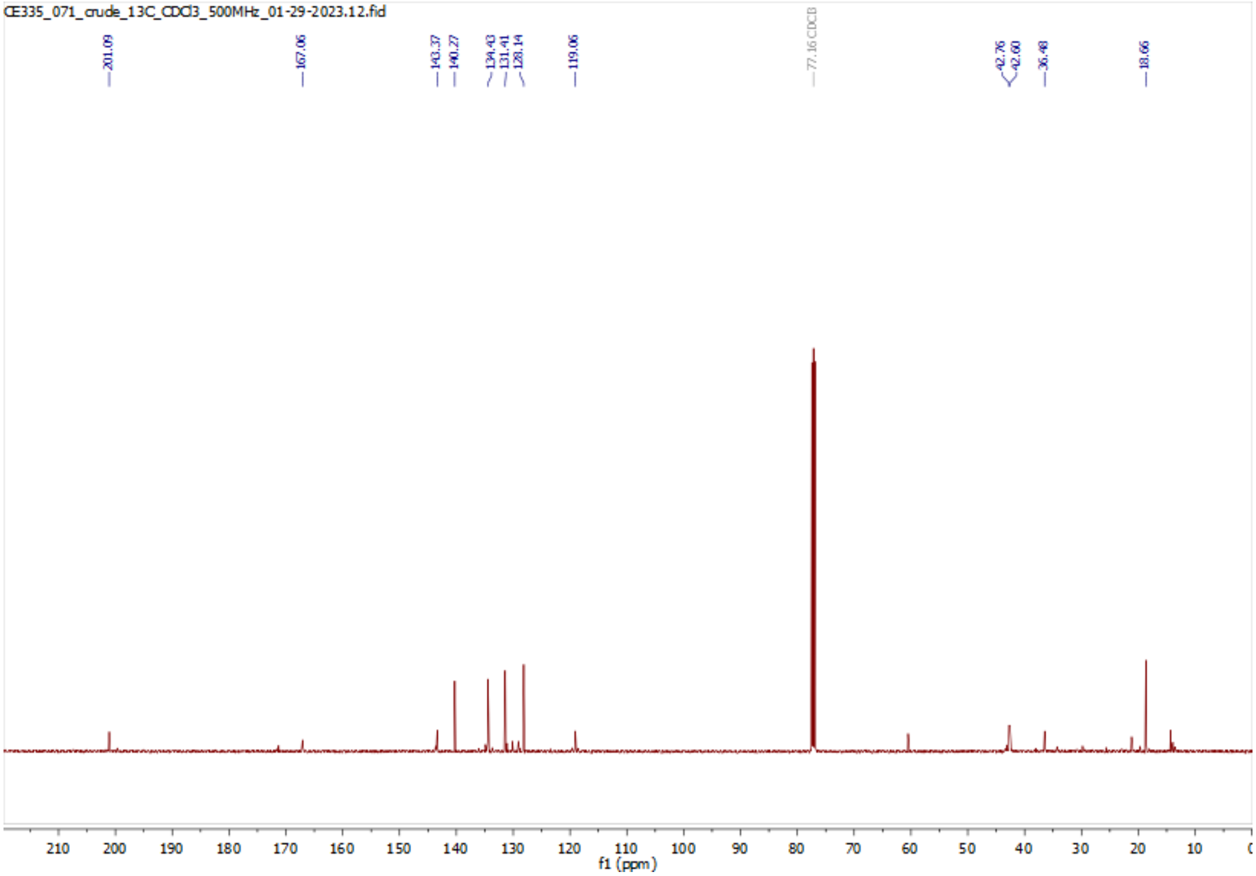
CE335_071_crude_13C_CDCl3_500MHz_01-29-2023.12.fid
201.09
167.06
143.37
140.27
134.43
131.41
128.14
119.06
77.16 CDCl3
42.76
42.60
36.48
18.66
210 200 190 180 170 160 150 140 130 120 110 100 90 80 70 60 50 40 30 20 10
f1 (ppm)

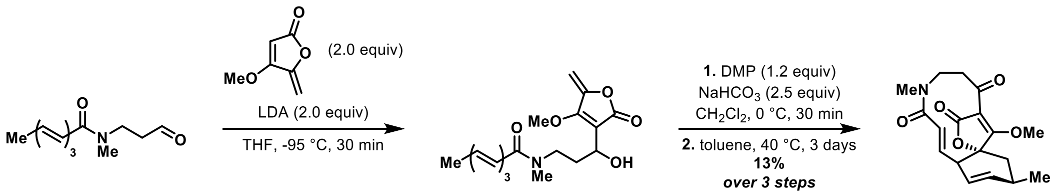

Diels-Alder product (15) A 50 mL round-bottom flask was charged with a stir bar, flame-dried under vacuum, and cooled under an atmosphere of argon. A solution of diisopropylamine (0.15 g, 1.45 mmol, 2 equiv) was made in toluene (1.5 mL) and ether (1.5 mL). The reaction flask was then cooled to -78 °C and allowed to stir for 5 minutes. Then a solution of n-butyllithium (0.80 mL, 1.45 mmol, 2.0 equiv) was added, and the reaction allowed to stir for 15 minutes. Then a solution of 4-methoxy-5-methylenefuran-2(5H)-one (0.18 g, 1.45 mmol, 2.0 equiv) in toluene (2.5 mL) and ether (2.5 mL) was added in a rapid dropwise manner and after 4 minutes a solution of (2E,4E,6E)-N-methyl-N-(3-oxopropyl)octa-2,4,6-trienamide **13** (0.15 g, 0.72 mmol, 1.0 equiv) in toluene (1 mL) and ether (1 mL) was added in a rapid dropwise fashion. The reaction was then allowed to stir for 30 minutes and then was quenched with a saturated, aqueous ammonium chloride solution (5 mL). The reaction was diluted with ethyl acetate, and the organic and aqueous layers were separated. The aqueous layer was extracted with ethyl acetate (3x), and then the organic layers were collected, washed with brine, dried (Na_2SO_4), and concentrated under reduced pressure. The crude mixture (0.25 g) would then be carried forward into the next step without purification. A 50 mL round-bottom flask would be charged with a stir bar, flame-dried under vacuum, and cooled under an atmosphere of argon. A solution of the crude mixture from the previous step would be made in dichloromethane (0.1 M, 7.6 mL) and the reaction vessel cooled to 0 °C. Sodium bicarbonate (0.16 g, 1.91 mmol, 2.5 equiv) would then be added in one portion followed by Dess-Martin periodinane (0.41 g, 0.92 mmol, 1.2 equiv). The reaction was allowed to stir for 30 minutes and then quenched with a saturated, aqueous sodium bicarbonate solution (10 mL). The organic and aqueous layers were separated, and the aqueous layer was extracted with dichloromethane (3x). The organic layers were collected, washed with brine, dried (Na_2SO_4), and then 20 mL of toluene was added to organic layer. The dichloromethane was then removed under reduced pressure and then the solution in toluene was allowed to stir for 3 days at 40 °C. At the end of 3 days, the toluene was removed under reduce pressure and the resulting crude product purified using automated chromatography to yield 0.03 g (13%) of a white solid. R_f = 0.22 (3:2 ethyl acetate: hexanes)

^{1}H NMR (500 MHz, $CDCl_3$) δ 6.61 (d, J = 16.3 Hz, 1H), 6.17 (dd, J = 16.2, 9.4 Hz, 1H), 5.94 (dt, J = 9.6, 3.2 Hz, 1H), 5.74 (dt, J = 9.6, 2.6 Hz, 1H), 4.38 (ddd, J = 15.7, 12.7, 3.2 Hz, 1H), 3.92 (s, 3H), 3.25 (dddt, J = 18.6, 11.6, 9.1, 3.0 Hz, 3H), 2.94 (s, 3H), 2.84 (ddd, J = 17.1, 12.8, 4.1 Hz, 1H), 2.39 (dtt, J = 10.2, 5.4, 2.9 Hz, 1H), 2.11 (dd, J = 14.1, 4.9 Hz, 1H), 1.65 (dd, J = 14.2, 9.5 Hz, 1H), 1.15 (d , J = 7.1 Hz, 3H).

^{13}C NMR (126 MHz, $CDCl_3$) δ 195.08, 182.15, 170.53, 167.61, 139.34, 138.22, 127.52, 125.82, 107.08, 89.01, 63.64, 46.41, 46.29, 42.72, 38.18, 32.61, 27.97, 20.29.

HRMS (*m/z*) $[M+H]^+$ calculated for $C_{18}H_{22}O_5N$ 332.14925, found 332.14923, $[M+Na]^+$ calculated for $C_{18}H_{21}O_5NNa$ 354.13119, found 354.13109, $[2M+H]^+$ calculated for $C_{36}H_{43}O_{10}N_2$ 663.29122, found 663.29106, $[2M+Na]^+$ calculated for $C_{36}H_{42}O_{10}N_2Na$ 685.27317, found 685.27279.

IR (Diamond-ATR, neat) $\tilde{\nu}_{max}$: 2952, 2932, 1744, 1677, 1616, 1446, 1351, 993.

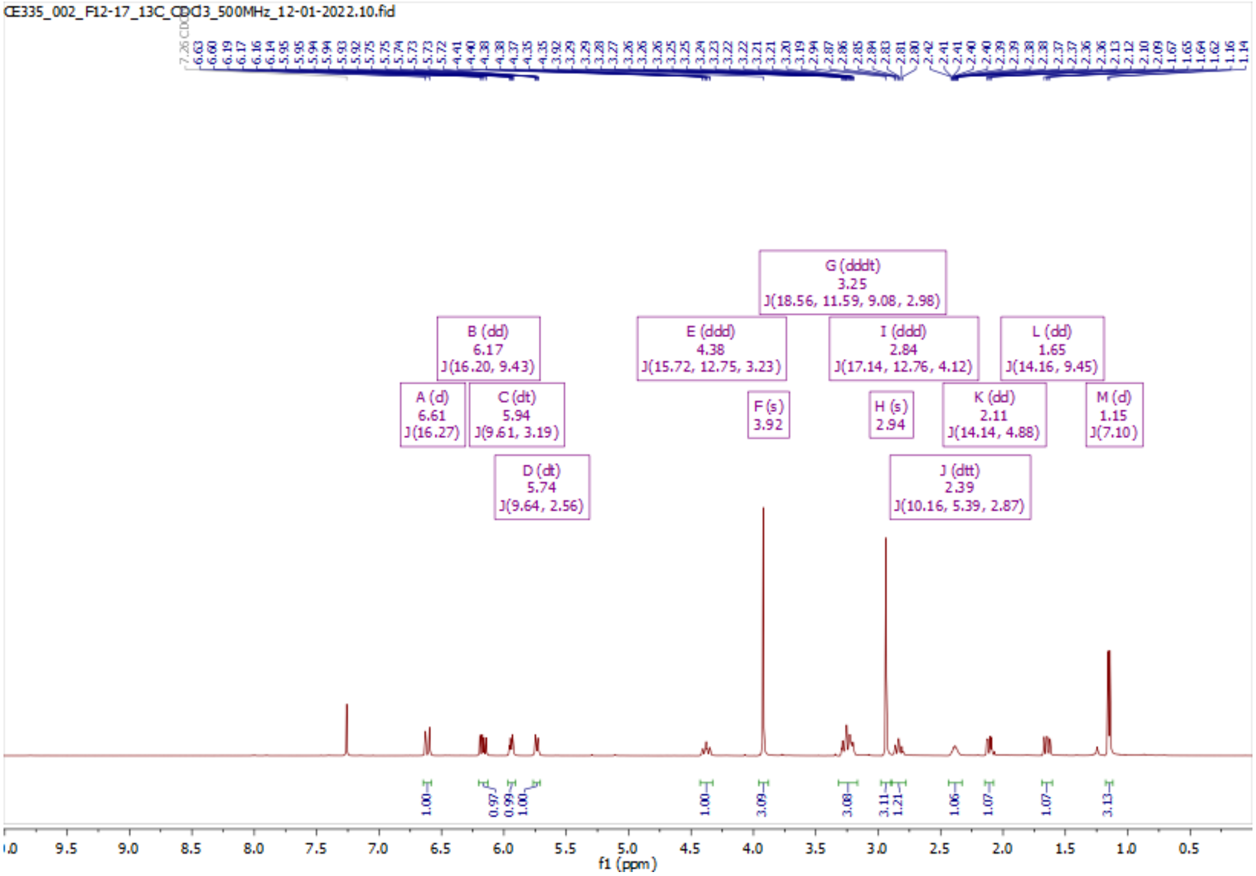
CE335_002_F12-17_13C_CDCl3_500MHz_12-01-2022.10.fid
G (dddt)
3.25
J(18.56, 11.59, 9.08, 2.98)
B (dd)
6.17
J(16.20, 9.43)
E (ddd)
4.38
J(15.72, 12.75, 3.23)
I (ddd)
2.84
J(17.14, 12.76, 4.12)
L (dd)
1.65
J(14.16, 9.45)
A (d)
6.61
J(16.27)
C (dt)
5.94
J(9.61, 3.19)
F (s)
3.92
H (s)
2.94
K (dd)
2.11
J(14.14, 4.88)
M (d)
1.15
J(7.10)
D (dt)
5.74
J(9.64, 2.56)
J (dtt)
2.39
J(10.16, 5.39, 2.87)
1.00
0.97
0.99
1.00
1.00
3.09
3.08
3.11
1.21
1.06
1.07
1.07
3.13
9.5
9.0
8.5
8.0
7.5
7.0
6.5
6.0
5.5
5.0
4.5
4.0
3.5
3.0
2.5
2.0
1.5
1.0
0.5
f1 (ppm)

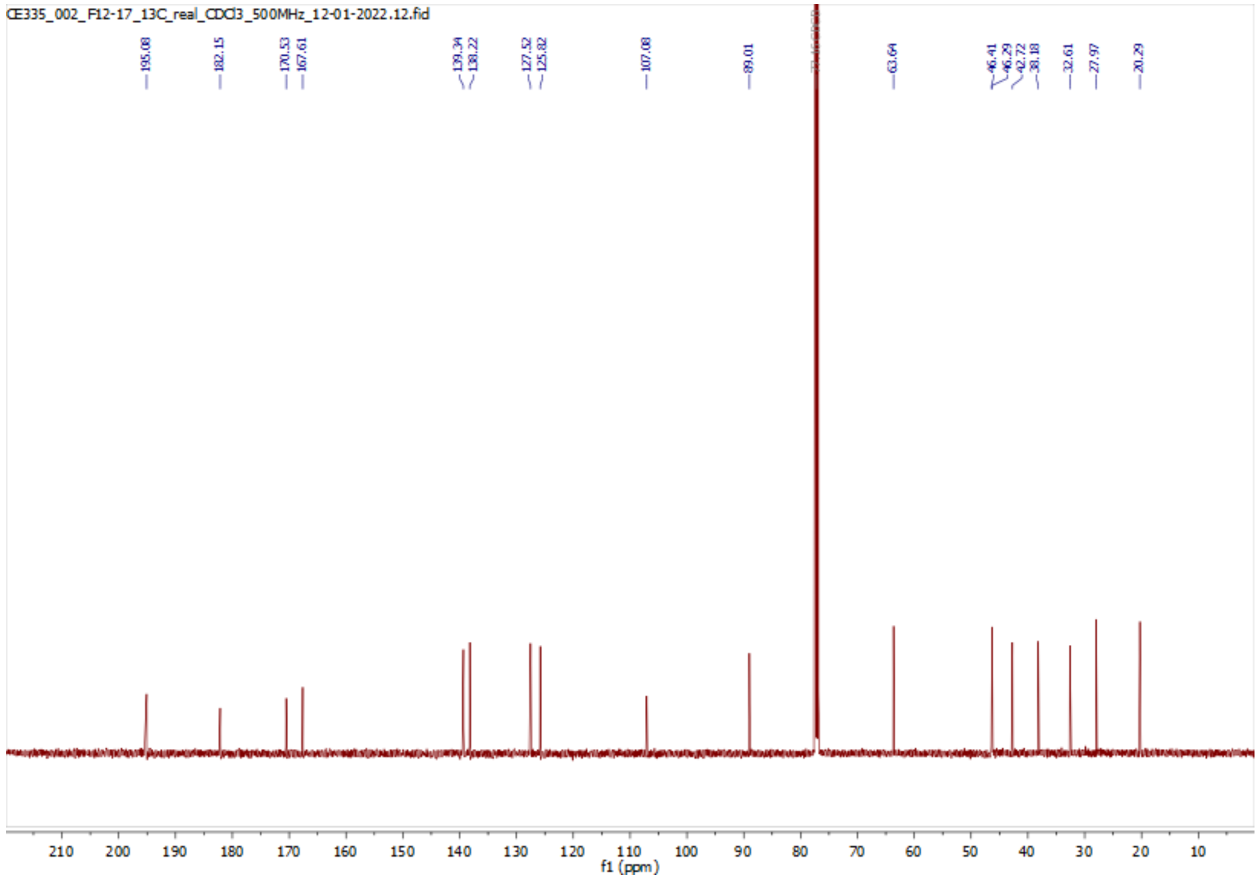

CE335_002_F12-17_13C_real_CDCl3_500MHz_12-01-2022.12.fid
195.08
182.15
170.53
167.61
139.34
138.22
127.52
125.82
107.08
89.01
63.64
46.41
46.29
42.72
38.18
32.61
27.97
20.29
210
200
190
180
170
160
150
140
130
120
110
100
90
80
70
60
50
40
30
20
10
f1 (ppm)

Crystallographic Data of 15 (for details see rs153)

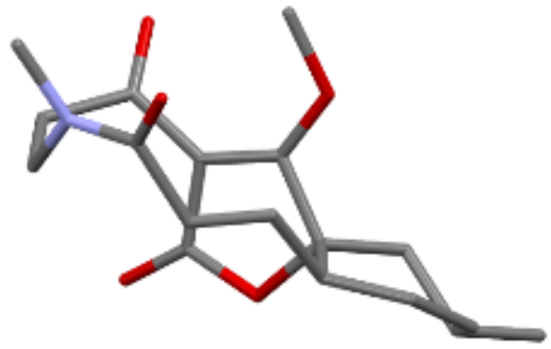

A colorless block-like specimen of $C_{18}H_{21}NO_{5.37}$, approximate dimensions 0.077 mm x 0.083 mm x 0.109 mm, was used for the X-ray crystallographic analysis. The X-ray intensity data were measured on a Bruker D8 VENTURE κ-geometry diffractometer system equipped with a Incoatec IµS 3.0 microfocus sealed tube (Cu Kα, λ = 1.54178 Å) and a multilayer mirror monochromator.

The total exposure time was 5.70 hours. The frames were integrated with the Bruker SAINT software package using a narrow-frame algorithm. The integration of the data using a monoclinic unit cell yielded a total of 35653 reflections to a maximum θ angle of 77.35° (0.79 Å resolution), of which 3423 were independent (average redundancy 10.416, completeness = 99.7%, Rint = 2.56%, Rsig = 1.57%) and 3372 (98.51%) were greater than 2σ(F2). The final cell constants of a = 7.01050(10) Å, b = 19.4681(4) Å, c = 11.9013(2) Å, β = 93.2770(10)°, volume = 1621.65(5) Å3, are based upon the refinement of the XYZ-centroids of 427 reflections above 20 σ(I) with 15.57° < 2θ < 136.7°. Data were corrected for absorption effects using the Multi-Scan method (SADABS). The ratio of minimum to maximum apparent transmission was 0.929. The calculated minimum and maximum transmission coefficients (based on crystal size) are 0.9130 and 0.9380.

The structure was solved and refined using the Bruker SHELXTL Software Package, using the space group P 1 21/c 1, with Z = 4 for the formula unit, C18H21NO5.37. The final anisotropic full-matrix least-squares refinement on F2 with 248 variables converged at R1 = 3.39%, for the observed data and wR2 = 8.63% for all data. The goodness-of-fit was 1.032. The largest peak in the final difference electron density synthesis was 0.268 e-/Å^3 and the largest hole was -0.236 e-/Å^3 with an RMS deviation of 0.039 e-/Å^3. On the basis of the final model, the calculated density was 1.382 g/cm^3 and F(000), 716 e$^-$.

Material is a co-crystal of olefin at C10-C11 and epoxide C10, C11, O6. Olefin:epoxide ratio refines to ca. 3:1. Restraints used to ensure reasonable atom shape and consistency through disordered section.

Table 1. Sample and crystal data for rs153.cif

**Identification code**	rs153
**Chemical formula**	C18H21NO5.37

**Formula weight**	337.31 g/mol
**Temperature**	100(2) K
**Wavelength**	1.54178 Å
**Crystal size**	0.077 x 0.083 x 0.109 mm
**Crystal habit**	colorless block
**Crystal system**	monoclinic
**Space group**	P 1 21/c 1
**Unit cell dimensions**	a = 7.01050(10) Å α = 90°<br>b = 19.4681(4) Å β = 93.2770(10)°<br>c = 11.9013(2) Å γ = 90°
**Volume**	1621.65(5) Å3
**Z**	4
**Density (calculated)**	1.382 g/cm3
**Absorption coefficient**	0.848 mm^{-1}
**F(000)**	716

Table 2. Data collection and structure refinement for rs153.cif

**Diffractometer**	Bruker D8 VENTURE κ-geometry diffractometer
**Radiation source**	Incoatec IμS 3.0 microfocus sealed tube (Cu Kα, λ = 1.54178 Å)
**Theta range for data collection**	4.36 to 77.35°
**Index ranges**	-8<=h<=8, -24<=k<=24, -15<=l<=14
**Reflections collected**	35653
**Independent reflections**	3423 [R(int) = 0.0256]
**Coverage of independent reflections**	99.7%
**Absorption correction**	Multi-Scan
**Max. and min. transmission**	0.9380 and 0.9130
**Structure solution technique**	direct methods
**Structure solution program**	SHELXT 2018/2 (Sheldrick, 2018)
**Refinement method**	Full-matrix least-squares on F^2
**Refinement program**	SHELXL-2018/3 (Sheldrick, 2018)

**Function minimized**	Σ w(F_o^2 - F_c^2)^2
**Data/restraints/parameters**	3423 / 42 / 248
**Goodness-of-fit on F^2**	1.032
**Δ/σ_{max}**	0.001
**Final R indices**	3372 data; I>2σ(I)
	R1 = 0.0339,wR2 = 0.0861
	all data
	R1 = 0.0342, wR2 = 0.0863
**Weighting scheme**	w=1/[$\sigma^2(F_o^2)+(0.0409P)^2+0.6807P$]
	where P=($F_o^2+2F_c^2$)/3
**Largest diff. peak and hole**	0.268 and -0.236 eÅ^{-3}
**R.M.S. deviation from mean**	0.039 eÅ^{-3}

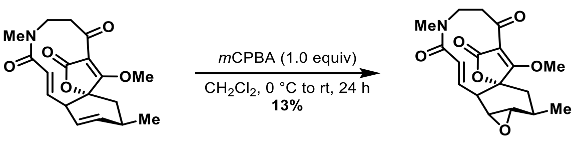

15 16

Epoxide (16) A 10 mL round-bottom flask was charged with a stir bar, flame-dried under vacuum, and cooled under an atmosphere of argon. A solution of the Diels-Alder product **15** (0.06 g, 0.18 mmol, 1.0 equiv) was made in dichloromethane (0.1 M, 1.8 mL). The reaction was then cooled to 0 °C, and after one minute a solution of *meta*-chloroperoxybenzoic acid (0.04 g, 0.18 mmol, 1.0 equiv) in dichloromethane (1.0 mL) was added dropwise. The reaction was allowed to stir for 12 hours and was then quenched with a saturated sodium thiosulfate solution. The organic and aqueous layers were separated, and the aqueous layer was extracted with dichloromethane (3x). The organic layers were then collected, washed with brine, dried (Na_2SO_4), and concentrated under reduced pressure. A pipet column was then used for purification to yield 0.01 g (13%) of a white solid. R_f = 0.22 (9:1 ethyl acetate: hexanes) which by NMR is a compounds and spectra are reported as observed for these compounds.

^{1}H NMR (600 MHz, $CDCl_3$) δ 6.62 (d, *J* = 16.6 Hz, 1H), 6.59 (d, *J* = 16.0 Hz, 1H), 6.48 (dd, *J* = 16.1, 9.8 Hz, 1H), 6.23 (dd, *J* = 16.3, 9.3 Hz, 1H), 4.35 (t, *J* = 13.4 Hz, 2H), 3.97 (s, 3H), 3.93 (s, 3H), 3.29 – 3.22 (m, 7H), 3.16 (d, *J* = 4.6 Hz, 1H), 3.14 (dd, *J* = 4.1, 2.2 Hz, 1H), 3.06 (t, *J* = 3.5 Hz, 1H), 2.95 (s, 4H), 2.94 (s, 4H), 2.90 – 2.81 (m, 3H), 2.36 (dtd, *J* = 13.7, 6.9, 4.4 Hz, 1H), 2.15 – 2.03 (m, 3H), 1.82 – 1.76 (m, 1H), 1.60 (dd, *J* = 14.6, 4.2 Hz, 1H), 1.24 (d, *J* = 7.5 Hz, 5H), 1.18 (d, *J* = 6.9 Hz, 4H).

^{13}C NMR (151 MHz, CDCl3) δ 195.42, 194.91, 181.57, 181.32, 170.20, 169.92, 167.37, 166.80, 136.82, 136.66, 128.26, 128.12, 107.70, 106.64, 87.29, 86.97, 63.92, 63.51, 57.35, 55.62, 53.45, 51.59, 47.11, 46.77, 46.38, 46.36, 42.72, 42.66, 34.77, 33.24, 32.71, 32.47, 29.85, 28.24, 26.17, 19.47, 18.56.

HRMS (*m*/*z*) $[M+H]^+$ calculated for $C_{18}H_{22}O_6N$ 348.14416, found 348.14414, $[M+Na]^+$ calculated for $C_{18}H_{21}O_6NNa$ 370.12611, found 370.12586, $[2M+H]^+$ calculated for $C_{36}H_{43}O_{12}N_2$ 695.28105, found 695.28098, $[2M+Na]^+$ calculated for $C_{36}H_{42}O_{12}N_2Na$ 717.26300, found 717.26261.

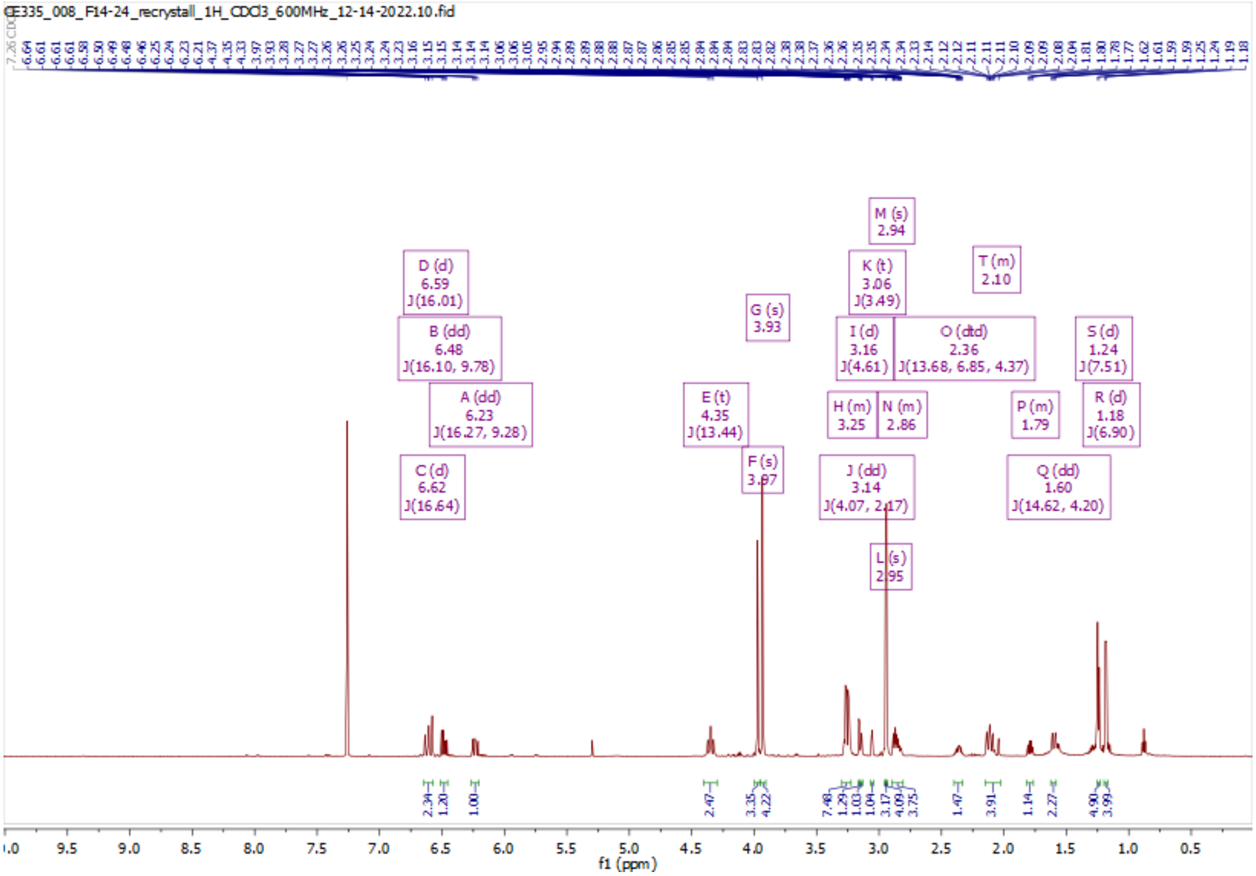
CE335_008_F14-24_recrystall_1H_CDCl3_600MHz_12-14-2022.10.fid
D (d) 6.59 J(16.01)
B (dd) 6.48 J(16.10, 9.78)
A (dd) 6.23 J(16.27, 9.28)
C (d) 6.62 J(16.64)
E (t) 4.35 J(13.44)
G (s) 3.93
F (s) 3.97
M (s) 2.94
K (t) 3.06 J(3.49)
I (d) 3.16 J(4.61)
O (dtd) 2.36 J(13.68, 6.85, 4.37)
H (m) 3.25
N (m) 2.86
J (dd) 3.14 J(4.07, 2.17)
L (s) 2.95
T (m) 2.10
P (m) 1.79
Q (dd) 1.60 J(14.62, 4.20)
S (d) 1.24 J(7.51)
R (d) 1.18 J(6.90)
f1 (ppm)

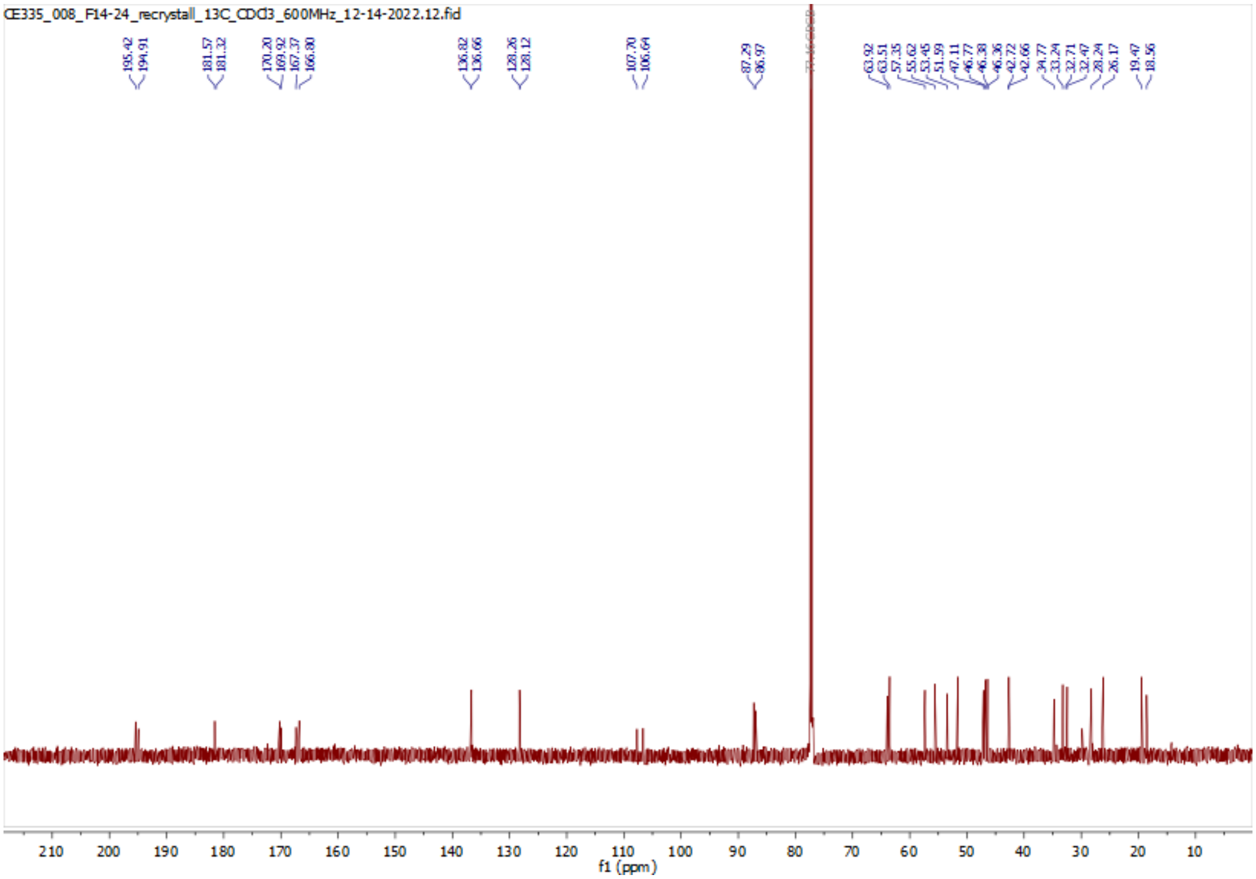
CE335_008_F14-24_recrystall_13C_CDCl3_600MHz_12-14-2022.12.fid
195.42
194.91
181.57
181.32
170.20
169.92
167.37
166.80
136.82
136.66
128.26
128.12
107.70
106.64
87.29
86.97
63.92
63.51
57.35
55.62
53.45
51.59
47.11
46.77
46.38
46.36
42.72
42.66
34.77
33.24
32.71
32.47
28.24
26.17
19.47
18.56
210
200
190
180
170
160
150
140
130
120
110
100
90
80
70
60
50
40
30
20
10
f1 (ppm)

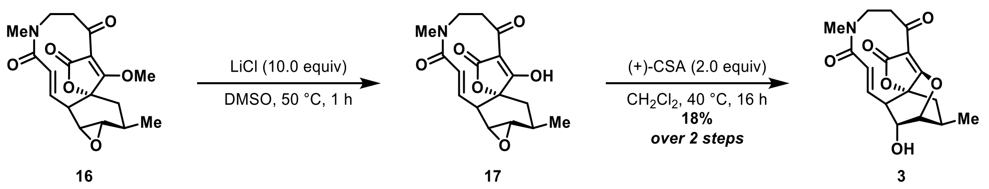

Lactam-abyssomicin (3) A solution of the vinylogous carbonate **16** (5.0 mg, 0.014 mmol, 1.0 equiv) in DMSO (0.1 M, 0.14 mL) was added to a 4 mL vial and lithium chloride (6.1 mg, 0.14 mmol, 10.0 equiv) was added in one portion. This was then heated to 50 °C and allowed to stir for one hour. After consumption of starting material was seen by LCMS, the reaction was cooled, lypholized overnight, and then filtered through silica (6% MeOH in CH_2Cl_2) to remove the remaining lithium chloride (acidifying before removal appears to add an equivalent of chloride to the molecule, seen by LCMS). Removal of the solvent yielded a white solid (4.8 mg) that was carried forward into the next step without further purification.

A solution of the vinylogous carbonic acid **17** (4.8 mg, 0.014 mmol, 1.0 equiv) was made using CH_2Cl_2 (0.06 M, 0.24 mL) in a 4 mL vial. Then a solution of (1S)-(+)-10-camphorsulfonic acid (3.4 mg, 0.0144 mmol, 1.0 equiv) was made in CH_2Cl_2 (0.06 M, 0.24 mL) and then was added to the reaction vial in one portion. Then the reaction was heated to 40 °C and the reaction allowed to stir overnight. The following day, the reaction was quenched with a saturated sodium bicarbonate solution and the organic layer was separated and filtered through a pipet of sodium sulfate. Then the aqueous was extracted two more times with CH_2Cl_2, each time the organic being filtered through the same pipet of sodium sulfate. Then the organic layers were concentrated under reduced pressure and purified via pipet chromatography to yield 0.85 mg of a white solid (18% over two steps). R_f = 0.13 (6% MeOH in CH_2Cl_2).

^{1}H NMR (700 MHz, $CDCl_3$) δ 6.53 (dd, *J* = 16.1, 6.7 Hz, 1H), 6.02 (d, *J* = 16.1 Hz, 1H), 4.59 (d, *J* = 2.6 Hz, 1H), 4.13 (s, 1H), 3.62 (dt, *J* = 16.7, 3.9 Hz, 1H), 3.55 (dd, *J* = 16.0, 11.4 Hz, 1H), 3.29 (dd, *J* = 13.0, 4.7 Hz, 1H), 3.18 (s, 1H), 2.99 (s, 3H), 2.83 (d, *J* = 14.3 Hz, 1H), 2.70 (d, *J* = 11.7 Hz, 1H), 2.66 (d, *J* = 10.8 Hz, 1H), 1.52 (dd, *J* = 12.6, 3.6 Hz, 1H), 1.22 (d, *J* = 7.2 Hz, 3H).

^{13}C NMR (176 MHz, $CDCl_3$) δ 194.86, 169.04, 166.85, 136.03, 125.67, 107.31, 83.74, 68.70, 49.97, 47.23, 40.14, 35.15, 34.28, 33.28, 29.85, 25.02, 18.98.

HRMS (*m/z*) $[M+H]^+$ calculated for $C_{17}H_{20}O_6N$ 334.12851, found 334.12858, $[M+Na]^+$ calculated for $C_{17}H_{19}O_6NNa$ 356.11046, found 356.11050, $[2M+H]^+$ calculated for $C_{34}H_{39}O_{12}N_2$ 667.24975, found 667.24989, $[2M+Na]^+$ calculated for $C_{34}H_{38}O_{12}N_2Na$ 689.23170, found 689.23178.

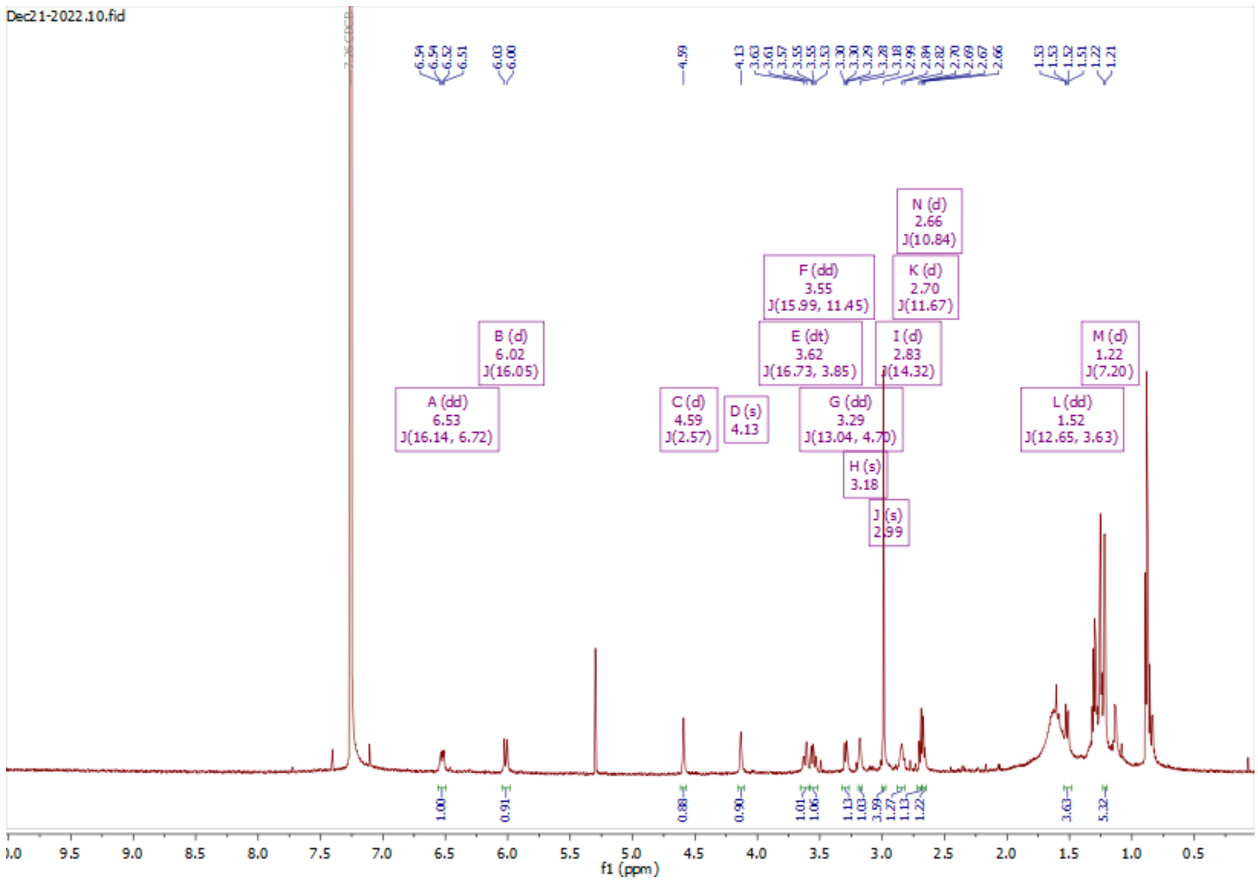

Dec21-2022.10.fid
A (dd) 6.53 J(16.14, 6.72)
B (d) 6.02 J(16.05)
C (d) 4.59 J(2.57)
D (s) 4.13
E (dt) 3.62 J(16.73, 3.85)
F (dd) 3.55 J(15.99, 11.45)
G (dd) 3.29 J(13.04, 4.70)
H (s) 3.18
I (d) 2.83 J(14.32)
J (s) 2.99
K (d) 2.70 J(11.67)
L (dd) 1.52 J(12.65, 3.63)
M (d) 1.22 J(7.20)
N (d) 2.66 J(10.84)
f1 (ppm)

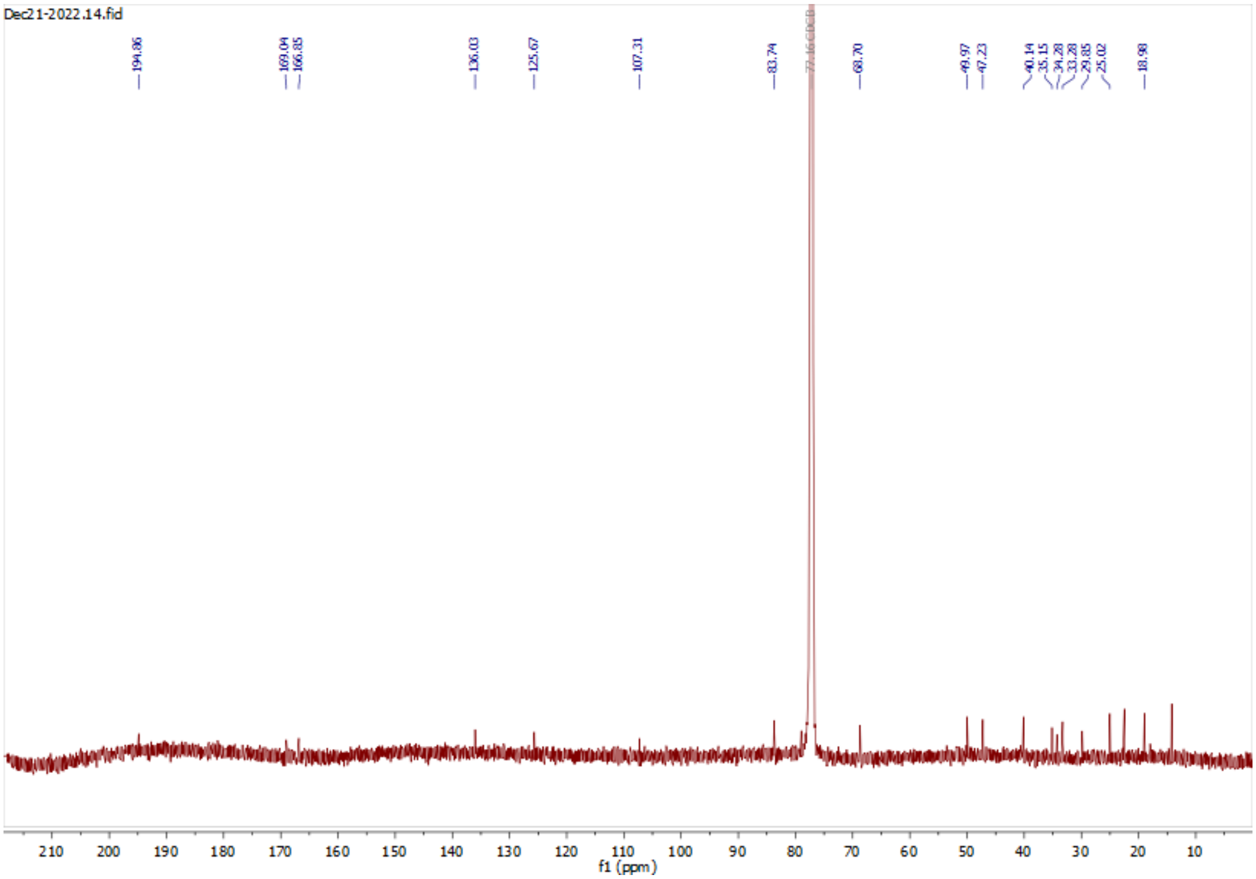

Dec21-2022.14.fid
194.86
169.04
166.85
136.03
125.67
107.31
83.74
77.16 CDCl3
68.70
49.97
47.23
40.14
35.15
34.28
33.28
29.85
25.02
18.98
210
200
190
180
170
160
150
140
130
120
110
100
90
80
70
60
50
40
30
20
10
f1 (ppm)

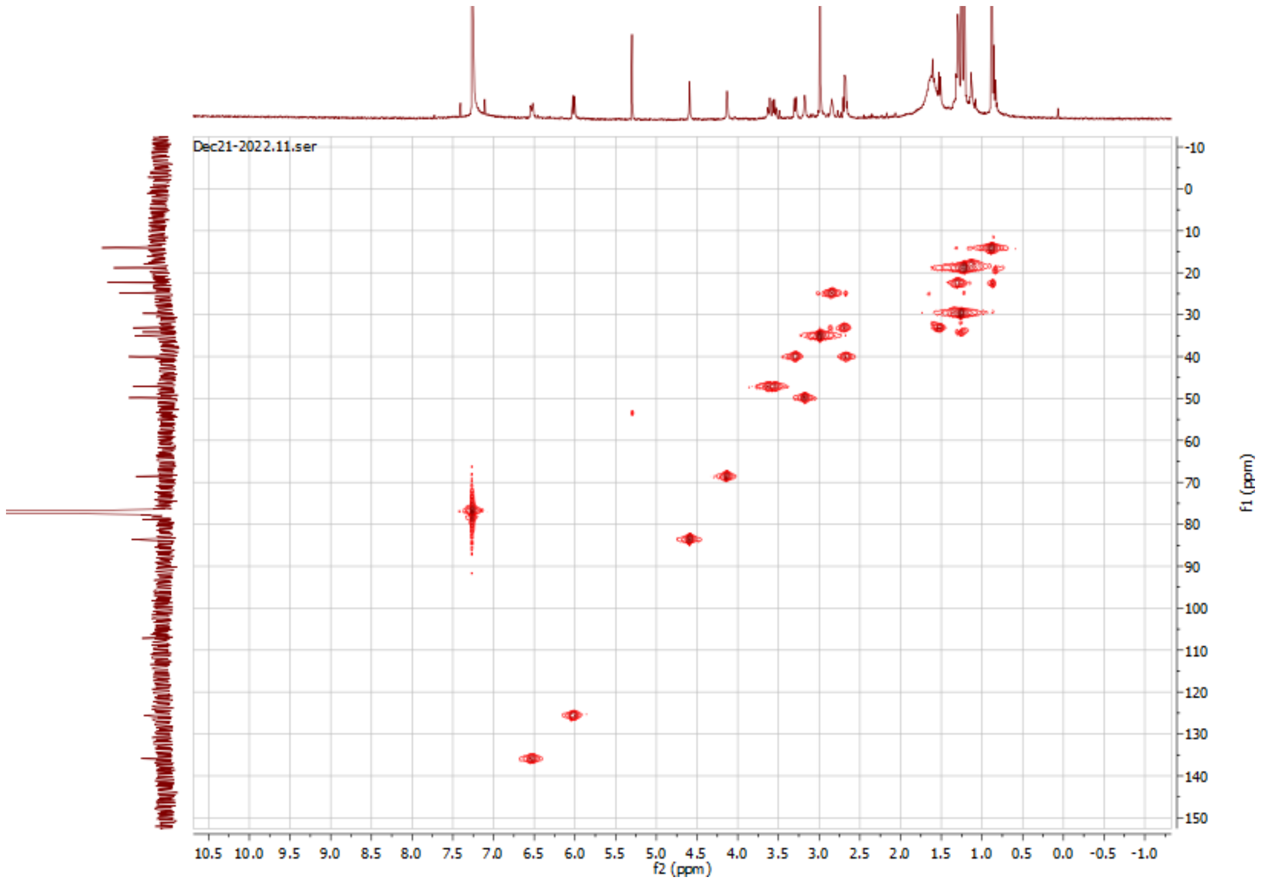
Dec21-2022.11.ser
f2 (ppm)
f1 (ppm)

Crystallographic Data of 3 (for details see sj.cif)

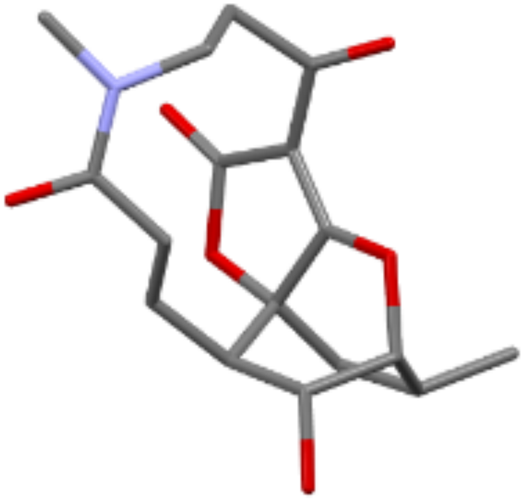

A colorless block-like specimen of $C_{17}H_{19}NO_6$, approximate dimensions 0.01 mm x 0.01 mm x 0.1 mm, was used for the X-ray crystallographic analysis. The X-ray intensity data were measured on a Bruker D8 VENTURE κ-geometry diffractometer system equipped with a Incoatec IμS 3.0 microfocus sealed tube (Cu Kα, λ = 1.54178 Å) and a multilayer mirror monochromator.

The total exposure time was 3.16 hours. The frames were integrated with the Bruker SAINT software package using a narrow-frame algorithm. The integration of the data using a monoclinic unit cell yielded a total of 8969 reflections to a maximum θ angle of 80.10° (0.8 Å resolution), of which 2811 were independent (average redundancy 2.71, completeness = 85.1%, Rint = 8.07%, Rsig = 8.04%) and 1766 (62.82%) were greater than 2σ(F2). The final cell constants of a = 10.071(3) Å, b = 6.773(2) Å, c = 11.780(4) Å, β = 110.27(2)°, volume = 753.8(4) $Å^3$, are based upon the refinement of the XYZ-centroids of 906 reflections above 20 σ(I) with 8° < 2θ < 160.196°. Data were corrected for absorption effects using the Multi-Scan method (SADABS). The ratio of minimum to maximum apparent transmission was 0.833. The calculated minimum and maximum transmission coefficients (based on crystal size) are 0.989 and 0.991.

The structure was solved and refined using the Bruker SHELXTL Software Package, using the space group P 1 21 1, with Z = 2 for the formula unit, $C_{17}H_{19}NO_6$. The final anisotropic full-matrix least-squares refinement on F^2 with 230 variables converged at R1 = 5.31%, for the observed data and wR2 = 13.72% for all data. The goodness-of-fit was 1.045. The largest peak in the final difference electron density synthesis was 0.203 $e^-/Å^3$ and the largest hole was -0.193 $e^-/Å^3$ with an RMS deviation of 0.052 $e^-/Å^3$. On the basis of the final model, the calculated density was 1.469 g/cm^3 and F(000), 352 e^-.

Table 1. Sample and crystal data for sj.cif

**Identification code**	sj
**Chemical formula**	$C_{17}H_{19}NO_6$
**Formula weight**	333.33 g/mol

**Temperature**	240 K
**Wavelength**	1.54178 Å
**Crystal size**	0.01 x 0.01 x 0.1 mm
**Crystal habit**	colorless needle
**Crystal system**	monoclinic
**Space group**	$P2_1$
**Unit cell dimensions**	a = 10.071(3) Å α = 90°<br>b = 6.773(2) Å β = 110.27(2)°<br>c = 11.780(4) Å γ = 90°
**Volume**	753.8(4) $Å^3$
**Z**	2
**Density (calculated)**	1.469 g/cm^3
**Absorption coefficient**	0.939 mm^{-1}
**F(000)**	352

Table 2. Data collection and structure refinement for sj.cif

**Diffractometer**	Bruker D8 VENTURE κ-geometry diffractometer
**Radiation source**	Incoatec IμS 3.0 microfocus sealed tube (Cu Kα, λ = 1.54178 Å)
**Theta range for data collection**	4.00 to 80.10°
**Index ranges**	-12<=h<=10, -8<=k<=7, -15<=l<=14
**Reflections collected**	8969
**Independent reflections**	2811 [R(int) = 0.0807]
**Coverage of independent reflections**	85.1%
**Absorption correction**	Multi-Scan
**Max. and min. transmission**	0.989 and 0.991
**Structure solution technique**	direct methods
**Structure solution program**	SHELXT 2018/2 (Sheldrick, 2018)
**Refinement method**	Full-matrix least-squares on F^2
**Refinement program**	Olex2 1.5 (Dolomanov et al., 2009)
**Function minimized**	$\Sigma w(F_o^2 - F_c^2)^2$

**Data/restraints/parameters**	2811 / 1 / 230
**Goodness-of-fit on F^2**	1.045
**Δ/σ_{max}**	0.000
**Final R indices**	1766 data; I>2σ(I)
	R1 = 0.0531,wR2 = 0.1134
	all data
	R1 = 0.0968, wR2 = 0.1372
**Weighting scheme**	$w=1/[\sigma^2(F_o^2)+(0.0569P)^2]$
	where $P=(F_o^2+2F_c^2)/3$
**Largest diff. peak and hole**	0.203 and --0.193 $eÅ^{-3}$
**R.M.S. deviation from mean**	0.052 $eÅ^{-3}$

Evaluation of Biological Activity

General Experimental

Methicillin-resistant *Staphylococcus aureus* (MRSA) strains were obtained from the American Type Culture Collection (ATCC: 43300, 33591) and colonies were grown on Mueller-Hinton broth (MHB) at 37 °C. Bacteria were kept in frozen stocks on glycerol at - 80 °C until use.

Mueller-Hinton broth (MHB, 211443-BD) was purchased from Fisher Scientific. Vancomycin (cat. # PZ0014) was purchased from Sigma-Aldrich. All assays were run in triplicate and repeated at least two separate times for MIC assays. All compounds were dissolved in molecular biology grade DMSO as 10 mM stock solutions. Optical densities were measured using a Thermo Scientific Genesys 20 spectrophotometer.

Broth microdilution method for determination of minimum inhibitory concentrations

As prescribed by the Clinical and Laboratory Standards Institute (CLSI) M07-A8, Vol. 29 (2) MRSA (ATCC 43300) and MRSA (ATCC 33591) are grown in Muller Hinton broth (MHB) for 6-8 h; this culture is then used to inoculate fresh MHB (5x105 CFU/mL). The resulting bacterial suspension is aliquoted (0.5 mL) into 1.5 mL Eppendorf tubes and testing compounds are added from a 10 mM DMSO stock to achieve the desired initial starting concentration (128 µg/mL). A known antibiotic or bioactive compound (Vancomycin, from a 10 mM DMSO stock) was used as a positive control. Inoculated media not treated with compound was used as the negative control. Serial dilution is performed through the entire plate, except for the control wells. The MIC is determined by micro broth dilution following the CLSI guidelines. The MIC is defined as the lowest concentration of antibiotic with no visible growth. The plate is sealed (using a plastic lid, not hermetic) and incubated under stationary conditions at 37 °C. After 16 h, MIC values are recorded as the lowest concentration of compound at which no visible growth of bacteria was observed, and results are recorded both by visual inspection and by using a 96-well plate reader.

References

1. Takeda, K.; Yano, S.; Sato, M.; Yoshii, E. Synthesis of the upper spirotetronic acid fragment of kijanolide *J. Org. Chem.* **1987**, *52*, 4135–4137

2. Naturale, G.; Lamblin, M.; Commandeur, C.; Felpin, F.; Dessolin, J. Direct C-H Alkylation of Naphthoquinones with Amino Acids Through a Revisited Kochi-Anderson Radical Decarboxylation: Trends in Reactivity and Applications *Eur. J. Org. Chem.* **2012**, *29*, 5774–5788
